# Mammalian plasma fetuin-B is a selective inhibitor of ovastacin and meprin metalloproteinases

**DOI:** 10.1038/s41598-018-37024-5

**Published:** 2019-01-24

**Authors:** Konstantin Karmilin, Carlo Schmitz, Michael Kuske, Hagen Körschgen, Mario Olf, Katharina Meyer, André Hildebrand, Matthias Felten, Sven Fridrich, Irene Yiallouros, Christoph Becker-Pauly, Ralf Weiskirchen, Willi Jahnen-Dechent, Julia Floehr, Walter Stöcker

**Affiliations:** 1 Institute of Molecular Physiology, Cell and Matrix Biology, Johannes Gutenberg University Mainz, 55099 Mainz, Germany; 2 Helmholtz Institute for Biomedical Engineering, Biointerface Laboratory, RWTH Aachen University, Medical Faculty, 52074 Aachen, Germany; 3 Institute of Biochemistry, CAU, 24118 Kiel, Germany; 4 Institute of Molecular Pathobiochemistry, Experimental Gene Therapy and Clinical Chemistry RWTH, 52074 Aachen, Germany

## Abstract

Vertebrate fetuins are multi-domain plasma-proteins of the cystatin-superfamily. Human fetuin-A is also known as AHSG, α_2_-Heremans-Schmid-glycoprotein. Gene-knockout in mice identified fetuin-A as essential for calcified-matrix-metabolism and bone-mineralization. Fetuin-B deficient mice, on the other hand, are female infertile due to zona pellucida ‘hardening’ caused by the metalloproteinase ovastacin in unfertilized oocytes. In wildtype mice fetuin-B inhibits the activity of ovastacin thus maintaining oocytes fertilizable. Here we asked, if fetuins affect further proteases as might be expected from their evolutionary relation to single-domain-cystatins, known as proteinase-inhibitors. We show that fetuin-A is not an inhibitor of any tested protease. In stark contrast, the closely related fetuin-B selectively inhibits astacin-metalloproteinases such as meprins and ovastacin, but not astacins of the tolloid-subfamily, nor any other proteinase. The analysis of fetuin-B expressed in various mammalian cell types, insect cells, and truncated fish-fetuin expressed in bacteria, showed that the cystatin-like domains alone are necessary and sufficient for inhibition. This report highlights fetuin-B as a specific antagonist of ovastacin and meprin-metalloproteinases. Control of ovastacin was shown to be indispensable for female fertility. Meprin inhibition, on the other hand, renders fetuin-B a potential key-player in proteolytic networks controlling angiogenesis, immune-defense, extracellular-matrix-assembly and general cell-signaling, with implications for inflammation, fibrosis, neurodegenerative disorders and cancer.

## Introduction

Control of proteolysis by specific proteinase inhibitors is a prerequisite for physiological homeostasis in health and disease ranging from fertilization, development, blood clotting and immune defense to cancer, Alzheimer’s disease, aging and cell death^1^. We have shown recently that fertilization in mammals is regulated through the inhibition of the astacin metalloproteinase ovastacin by fetuin-B, a hepatic plasma protein^2–4^.

Ovastacin is expressed in the oocyte^5^ and stored in cortical granules beneath the plasma membrane (oolemma) of the unfertilized egg, which is surrounded by an extracellular matrix termed zona pellucida^6–8^. Sperm penetration triggers the bulk release from cortical granules of ovastacin, which cleaves the zona pellucida protein 2 (ZP2) at a specific site (167LA*DE170)^6^. This limited proteolysis of a major component of the zona pellucida is thought to impair sperm-zona interactions, and causes definitive ‘hardening’ of the zona pellucida^6,9,10^.

Fetuin-A and fetuin-B are paralogous plasma proteins of the cystatin superfamily^11^. Many cystatins have been identified as inhibitors of papain-like cysteine proteinases^12^. Hence, the discovery of fetuin-B as an indispensable nanomolar inhibitor of the metalloproteinase ovastacin was unexpected^2^. However, the recent discovery that the caspase-like cysteine proteinase legumain can be inhibited by cystatins has broadened our understanding of enzyme inhibition in that evolutionary distinct families of proteases can be targeted by the same type of inhibitors^13,14^. Fetuin-A (FETUA/AHSG), fetuin-B (FETUB), histidine-rich glycoprotein (HRG) and kininogen (KNG)^15^ are circulating hepatic glycoproteins containing two (fetuins, HRG) or three (KNG) cystatin-like domains, and additional domains of different length and often unknown function. In contrast to single-domain cystatins^16^ and kininogens^17^, mammalian fetuin-A is incapable of inhibiting cysteine proteinases^15,18^, but was identified as an important regulator of mineralized matrix instead^19,20^. Depending on the genetic background, mice with a disrupted fetuin-A gene^21^ suffer from severe ectopic calcification^22^ or bone dysplasia^20^. By contrast, fetuin-B deficient mice are female infertile due to premature zona pellucida ‘hardening’, which is caused by ovastacin activity in unfertilized eggs. In wild type mice the activity of spuriously released ovastacin is inhibited by micromolar concentrations of the plasma protein fetuin-B in the follicular fluid until after fertilization, when complete degranulation of oocytes releases large amounts of ovastacin, which override the fetuin-B inhibition^2,3^.

Fetuin-B inhibition of ovastacin activity was the first description of a mammalian plasma protein acting as a specific high-affinity inhibitor of an astacin metalloproteinase. We asked, if further physiological target peptidases for fetuin-B exist. In humans and mice, six genes encode astacin proteinases^23^. Besides ovastacin, these comprise the bone morphogenetic protein (BMP-1), the mammalian tolloid-like proteinases (mTLL), and the meprin proteinases^23–25^. BMP-1 and mTLL proteinases are involved in the assembly and remodeling of the extracellular matrix and they are crucial for dorsoventral axis formation during embryogenesis^26^. Meprin α and meprin β localize to apical epithelial membranes or to the pericellular space and the extracellular matrix. Meprins cleave procollagens^27,28^ and activate other cell surface proteases, e.g. a disintegrin and metalloprotease 10 (ADAM10)^29,30^. Furthermore meprins have β-secretase activity in cleaving the amyloid precursor protein (APP)^29,31^ and they cleave interleukin-1β, interleukin 18, interleukin 6-receptor, and many other substrates^30,32–35^, suggesting functions in angiogenesis^36^, cancer^37^, inflammation, fibrosis and neurodegenerative diseases^25,38^.

Before embarking on the study of interactions of plasma fetuins with potential target enzymes we needed to deepen the understanding of the biochemical properties of ovastacin, the first mammalian peptidase, that turned out to be a physiological target of fetuin-B during fertilization^2^. Interestingly, oocytes contain two variants of ovastacin protein, a 44 kDa form and a 29 kDa form - presumably inactive zymogen (pro-ovastacin) and mature enzyme (ovastacin), respectively^4^.

Here we show that pro-ovastacin is converted to active ovastacin by proteinases with trypsin-like specificity. Fetuin-B acts as a selective inhibitor in the extracellular space, for which at present only three target enzymes are known in mammals, namely ovastacin, meprin α and meprin β. Fetuin-B fails to inhibit aspartate, serine or cysteine proteinases, or other metalloproteinases such as MMPs or ADAMs, or BMP-1/tolloid-like astacin metalloproteinases, attesting a high specificity of inhibition. Neither glycosylation nor the expression system affected fetuin-B activity. Fetuin-A was found to inhibit none of the above proteinases.

## Results

### Activation and functional properties of ovastacin

**Figure 1:**
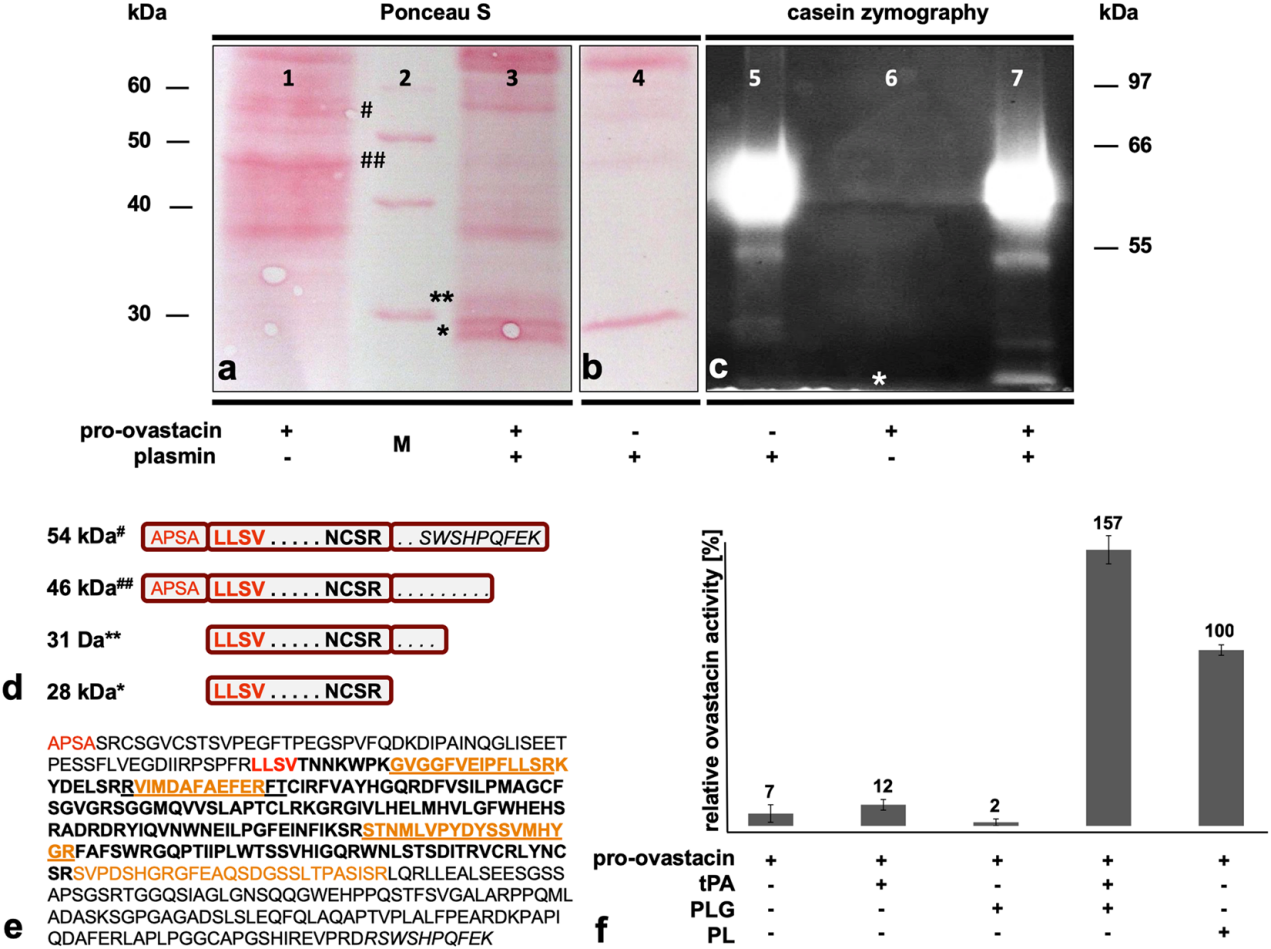
Activation of pro-ovastacin by human plasmin or by the combination of plasminogen and tissue-type plasminogen activator. (**a**,**b**) Ponceau S staining of pro-ovastacin (5.4 µM; 18 µg/lane) following activation with plasmin, separation by SDS-PAGE (12% acrylamide) and transfer onto PVDF membrane. The 54 kDa fragment (#lane 1) comprises the full length pro-ovastacin starting with the N-terminal sequence 24APSA. The 46 kDa fragment (##lane 1) is C-terminally truncated and has the same N-terminus. Edman degradation of the 28 kDa fragment (*lane 3) yielded the N-terminal sequence 86LLSV. (**c**) Casein-zymography showing plasmin activity (60 kDa) and plasmin-activated ovastacin (28 kDa lane 7), and absence of activity in pro-ovastacin (lane 6). (**d**) Cartoon showing the composition of pro-ovastacin (#, ##) and ovastacin (*, **) variants produced by proteolytic processing; top line, full-length Strep-tagged pro-ovastacin. (**e**) Amino acid sequence of pro-ovastacin with C-terminal Strep-tag (*italics*); catalytic domain in bold face. Protein mass spectrometry of the 28 kDa and the 31 kDa fragments (* and ** in a, lane 3) yielded underlined and orange colored peptides, respectively. (**f**) 600 nM pro-ovastacin was treated with t-PA (6:1), PLG (10:1) or PLG/t-PA (10:1:1) for 30 min at 37 °C before addition of 10 mM Pefabloc^®^. PL = activation by active plasmin. 100% ovastacin activity corresponds to a turnover rate of 4.6 nM/s Ac-RE(Edans)-DR-Nle-VGDDPY-K(Dabcyl)-NH_2_. The error bars indicate the standard deviation of duplicate measurements.

The pro-ovastacin zymogen was heterologously expressed in insect cells in order to study the mechanism of zymogen activation. In this expression system, pro-ovastacin undergoes partial proteolysis at its C-terminal non-catalytic domain liberating peptides migrating at about 54 kDa and 46 kDa in reducing SDS-PAGE (# and ## in Fig. 1a). Edman-degradation of both fragments yielded the sequence 24APSA, representing the predicted N-terminus of pro-ovastacin. Bioinformatic analysis based on the X-ray-crystal structure of pro-astacin^39^ had suggested that trypsin-like serine proteinases potentially trigger the maturation of pro-ovastacin *in vivo*. Hence, we performed activation studies with tissue-type plasminogen activator (t-PA), which is present in oocytes^40^, and with plasmin, which is present in follicular and oviductal fluid^41,42^.

Treatment of recombinant pro-ovastacin with active plasmin liberated peptides of M_*r*_ 28 kDa and 31 kDa (* and ** in Fig. 1a). Edman-sequencing of the 28 kDa cleavage product revealed the sequence 86LLSV representing the predicted amino-terminal sequence for mature ovastacin^39^. MALDI-TOF-TOF mass spectrometry of the 28 kDa* fragment from the SDS-PAGE identified peptides exclusively corresponding to the catalytic domain of ovastacin (Fig. 1d,e). In contrast, the 31 kDa fragment (**) contained also a peptide mapping to the C-terminal domain (Fig. 1d,e). Plasmin-treated pro-ovastacin cleaved casein in zymography (* in Fig. 1c). While t-PA alone failed to cleave pro-ovastacin, an equimolar mixture of t-PA and plasminogen cleaved pro-ovastacin better than did plasmin alone (Fig. 1f).

### Varying protein expression system does not alter inhibitory potency of mouse fetuin-B

Mammalian fetuin-A and fetuin-B were alternatively expressed in adenovirus transduced COS-7 cells^2^, in plasmid transfected CHO cells and in baculovirus transduced High Five insect cells. Molecular masses of the recombinant fetuins varied according to their degree of N-linked glycosylation. Glycan analysis of the recombinant proteins by lectin blotting demonstrated that COS-7 cell-derived fetuins had complex glycosylation with terminal sialic acid, CHO cell-derived fetuins had complex glycosylation largely devoid of terminal sialylation (cf. Supplementary Fig. S1), and High Five insect cell-derived fetuins typically had mannose-terminated N-glycans, with little sialylation. Moreover, fetuin-B was also expressed in CHO lec1 cells, which lack complex and hybrid-type glycosylation and in presence of tunicamycin, which prevents regular glycosylation. All these differences in glycosylation did not alter the inhibitory potency (cf. Supplementary Fig. S2). Regardless of the cell lines employed for recombinant expression, fetuin-A did not inhibit ovastacin, while fetuin-B from all cell lines strongly inhibited ovastacin (cf. Supplementary Fig. S2). Moreover, bacterially expressed, fish (carp) fetuin-A, truncated behind the two cystatin-like domains had undiminished inhibitory potency.

### Fetuin-B, but not fetuin-A inhibits substrate cleavage by mouse ovastacin, human meprin α and β, zebrafish nephrosin and crayfish astacin

**Figure 2:**
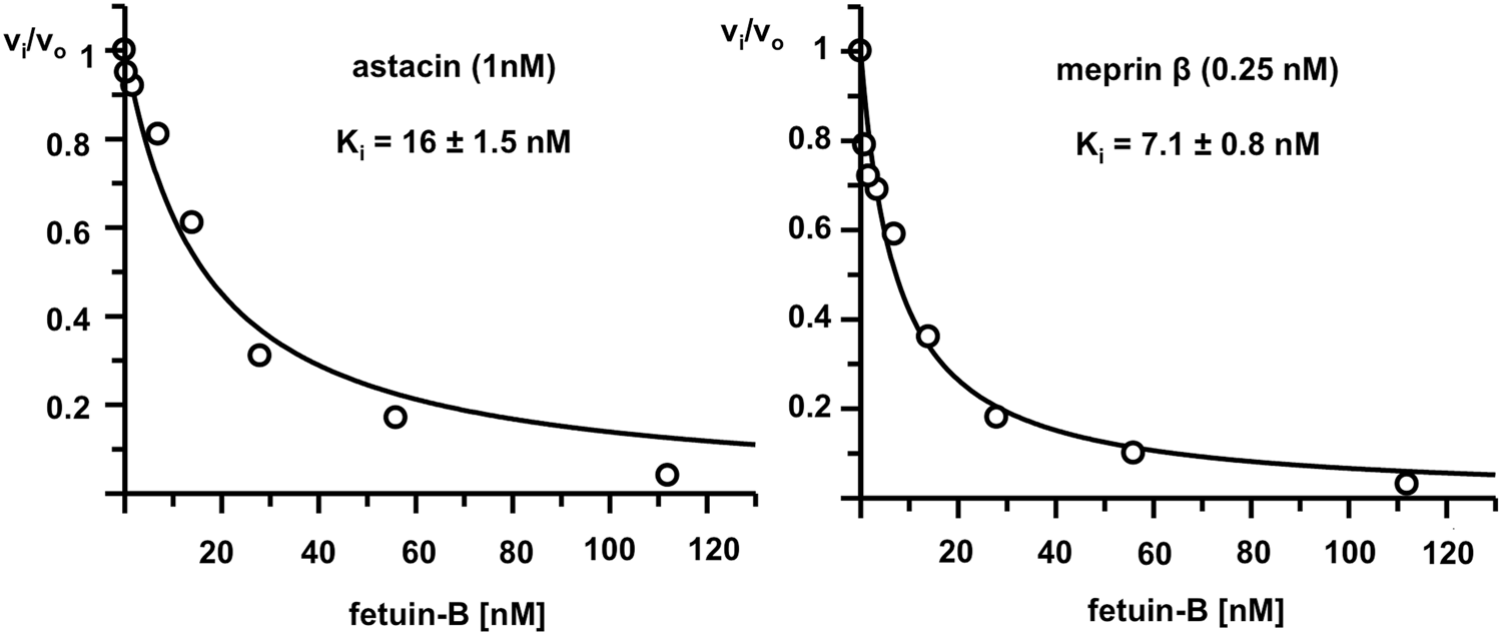
Inhibition of astacin and meprin β by fetuin-B. Astacin (left) or meprin β (right) were incubated with increasing amounts of fetuin-B and initial velocities (v_o_ in absence and v_i_ in presence of fetuin-B) of astacin, and meprin β activity was determined using the fluorescent substrates 400 µM Dns-PKRAPWV or 20 µM Ac-R-E(Edans)-DR-Nle-VGDDPY-K(Dabcyl)-amide, respectively.

Knowing that mammalian fetuin-B was a potent inhibitor of ovastacin, we tested the inhibitory potential of this plasma protein against other mammalian astacin metalloproteinases. Of these, only meprin α and meprin β (Fig. 2) were inhibited by mouse fetuin-B with similar potency like ovastacin, whereas the BMP-1/tolloid-proteases BMP-1 and TLL-2 were not inhibited (Table 1). On the other hand, there was potent inhibition of non-mammalian astacins such as zebrafish nephrosin and crayfish astacin (Table 1, Fig. 2). In contrast to fetuin-B, fetuin-A did not inhibit any of the astacins tested (Table 1).

**Table 1:** Inhibition of proteinases by recombinant mouse fetuin-A and fetuin-B.

Proteinase	[enzyme]	[S]	Concentration range [fetuin-A; fetuin-B]	Ki [nM]; IC50 [nM] fetuin-A	Ki [nM]; IC50 [nM] fetuin-B
meprin α	0.25 nM	20 µM	3.50 nM–100 nM	n.i.	Ki 33 ± 2.4
meprin β	0.25 nM	20 µM	0.90 nM–100 nM	n.i.	Ki 7 ± 0.8
astacin	1.00 nM	400 µM	0.20 nM–900 nM	n.i.	Ki 16 ± 1.5
ovastacin	320.00 nM	21 µM	0.40 nM–1200 nM	n.i.	IC50 18 ± 1.2
nephrosin	10.00 nM	12 µM	0.02 nM–1 µM	n.i.	IC50 0.6 ± 0.1
TLL2	180.00 nM	20 µM	0.20 nM–2 µM	n.i.	n.i.
BMP1	10.00 nM	24 µM	0.20 nM–20 µM	n.i.	n.i.
ADAM10	38.00 nM	20 µM	1.0 µM	n.i.	n.i.
MMP-2	20.00 nM	20 µM	0.1 µM–20 µM	n.i.	n.i.
MMP-8	25.00 nM	20 µM	0.20 nM–20 µM	n.i.	n.i.
MMP-9	20.00 nM	20 µM	0.20 nM–20 µM	n.i.	n.i.
MMP-13	3.90 nM	20 µM	0.20 nM–20 µM	n.i.	n.i.
trypsin	5 nM	100 µM	100 nM–1 µM	n.i.	n.i.
chymotrypsin	19 nM	100 µM	100 nM–1 µM	n.i.	n.i.
legumain	10.00 nM	50 µM	100 nM–1 µM	n.i.	n.i.
papain	140.00 nM	50 µM	150 nM–300 nM	n.i.	n.i.
cathepsin B	10.00 nM	20 µM	10 nM–1 µM	n.i.	n.i.
cathepsin K	200.00 nM	320 µM	100 nM–1 µM	n.i.	n.i.
cathepsin S	50.00 nM	320 µM	50 nM–1 µM	n.i.	n.i.
cathepsin D	40.00 nM	30 µM	10.0 nM–1 µM	n.i.	n.i.

Proteinase activity assays were performed with fluorescent substrates as detailed in the Methods section. Generally, we used seven or nine different duplicate concentrations of fetuins A and B in the indicated concentration range. Kinetic parameters were determined by fitting the data to the equation for tight binding inhibitors according to^71^. The standard errors of the fit are indicated. Due to the detection limits of substrate hydrolysis at low enzyme concentrations, it was not possible to determine a K_i_-value for ovastacin and nephrosin. n.i.: no inhibition.

### Fetuin-A and fetuin-B do not show any inhibitory effect against other metzincins or proteinases of other mechanistic classes

**Figure 3:**
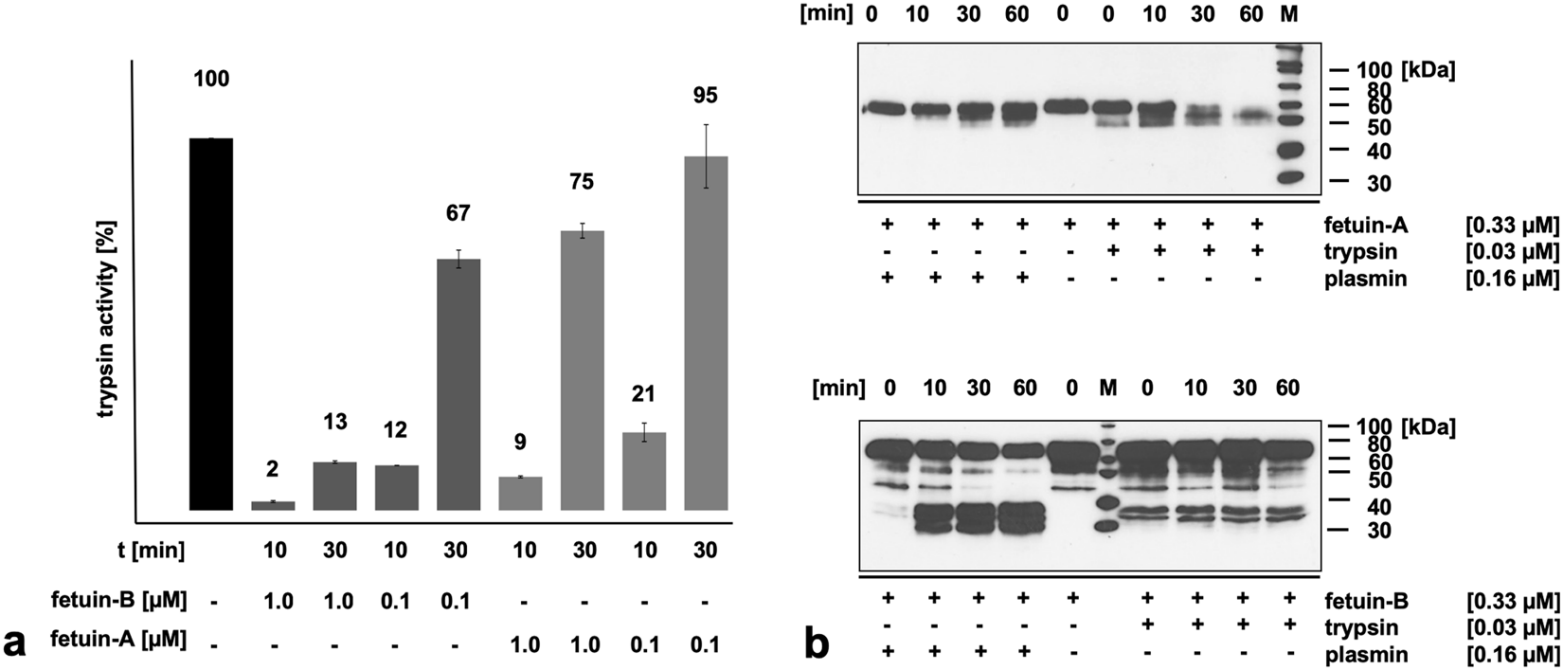
Apparent temporary inhibition of trypsin activity by fetuins and their cleavage by trypsin and plasmin. (**a**) 5 nM trypsin was pre-incubated with fetuin-A or fetuin-B for 10 min and 30 min at 37 °C. After the incubation proteolytic activity was measured with 100 µM fluorogenic substrate Boc-FSR-MCA at 37 °C. 100% activity corresponds to a substrate turnover rate of 10 nM/s. (**b**) Trypsin and plasmin were incubated with fetuin-A or fetuin-B for the indicated times at 37 °C. The reaction was stopped by boiling in SDS sample buffer for five minutes. Fetuin fragments were detected by immunoblot using homemade polyclonal rabbit antibodies directed against mouse fetuin-A or mouse fetuin-B.

To study whether fetuins inhibited further proteinases, we tested metzincin superfamily metalloproteinases ADAM10, MMP-2, MMP-9, MMP-8, MMP-13, serine proteinases chymotrypsin, trypsin, plasmin, tissue-type plasminogen activator, cysteine proteinases papain, cathepsins B, cathepsins S, cathepsins K, legumain, and the aspartate proteinase cathepsins D. None of these enzymes was inhibited by fetuin-A or fetuin-B (Table 1). A transient inhibition of trypsin activity by fetuin-A and fetuin-B was observed, which vanished after pre-incubation of enzyme and fetuin-A for about 30 min (Fig. 3a).

As shown in Fig. 3b, serine proteinases like trypsin and plasmin cleave fetuin-A and fetuin-B into large fragments of 65 kDa and 55 kDa. Surprisingly, fetuin-B cleaved by trypsin and chymotrypsin was still active as an ovastacin inhibitor (Supplementary Fig. S3), suggesting that limited proteolysis left intact the tertiary structure required for protease inhibition. Supplementary Fig. S3 demonstrates that on non-reducing SDS-gel electrophoresis trypsin- and chymotrypsin-cleaved fetuin-B indeed migrated as single bands indicating that the molecule remained disulfide-bonded and functionally intact despite proteolytic cleavage.

### Cleavage of fetuin-A, but not fetuin-B by astacin metalloproteinases

**Figure 4:**
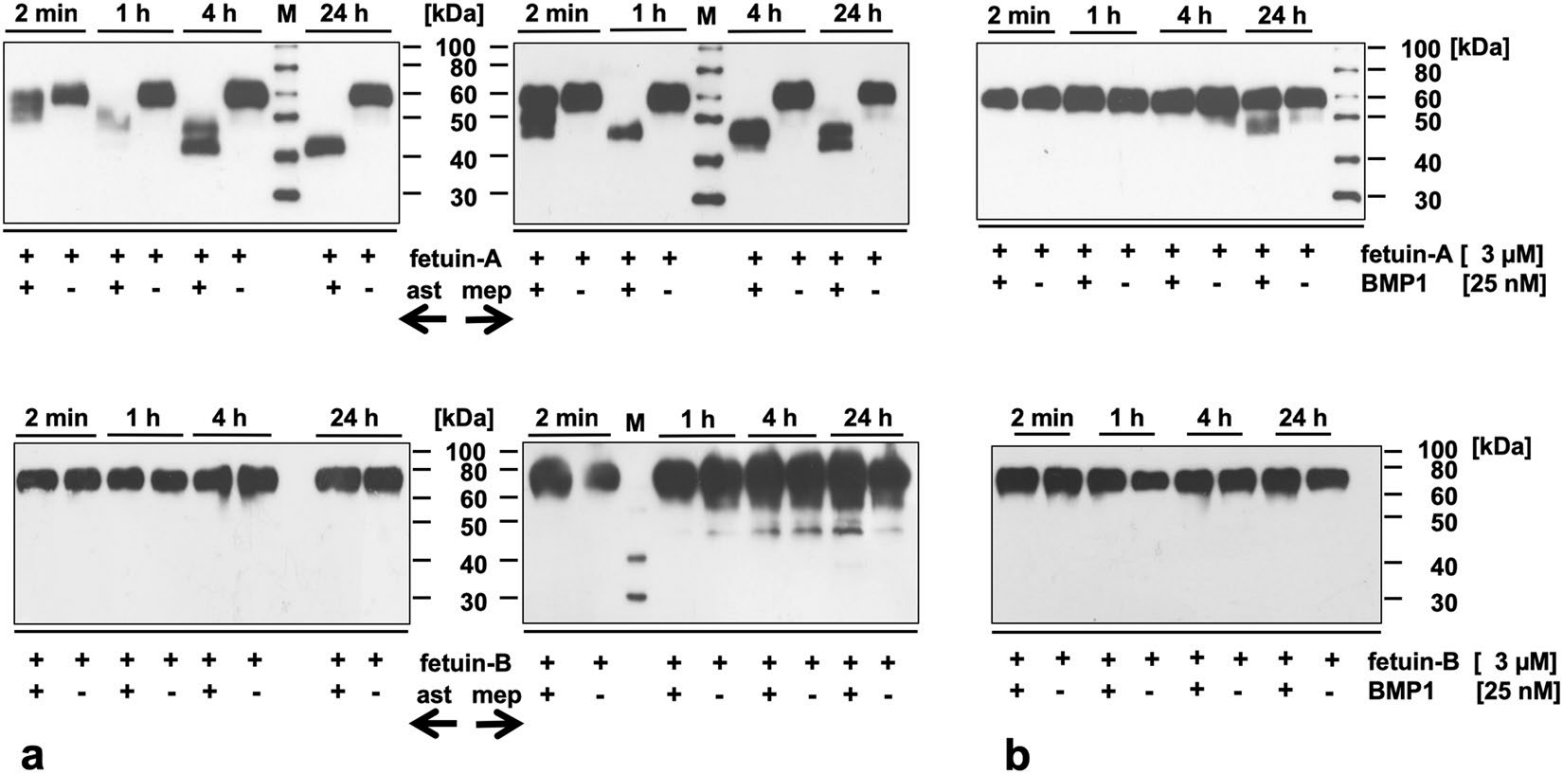
Susceptibility of fetuin-A and fetuin-B to cleavage by astacins. (**a**) Astacin (ast, left ←) and meprin β (mep, right →) (25 nM) were incubated with fetuin-A or fetuin-B (3 µM) at 37 °C for up to 24 h. Fetuin fragments were detected by immunoblot using homemade polyclonal rabbit antibodies directed against mouse fetuin-A or mouse fetuin-B. (**b**) BMP1 was incubated with fetuin-A or fetuin-B at 37 °C for up to 24 h. Fetuin fragments were detected by immunoblot using homemade polyclonal rabbit antibodies directed against mouse fetuin-A or mouse fetuin-B.

Next, we analyzed astacin-mediated proteolysis of fetuin-A and fetuin-B. Astacin (Fig. 4a), meprin β (Fig. 4a) and meprin α^43^ readily cleaved the 60 kDa fetuin-A liberating fragments of about 40 kDa within one to 24 hours. An exception was BMP-1, which cleaved only a minute portion of the starting material yielding a fragment of about 50 kDa within 24 hours (Fig. 4b). Fetuin-B cleavage products could not be detected upon incubation with astacin proteinases (Fig. 4). These results confirm that fetuin-A is a substrate of astacin metalloproteinases, while fetuin-B is an inhibitor of astacin, which is not cleaved at the molar ratios of the assay.

## Discussion

Cystatins are well established inhibitors of papain-like cysteine proteinases, and therefore it was hypothesized that fetuins should likewise inhibit cysteine proteinases. The fact that plasma kininogens containing three cystatin-like domains indeed inhibited cysteine proteinases^17^ supported this hypothesis. Here we showed, however, that neither fetuin-A nor fetuin-B are classical cystatin-like thiol proteinase inhibitors, but that fetuin-A was a substrate, not an inhibitor for proteinases of the major proteinase classes serine, cysteine, aspartate, and metallo. In contrast, fetuin-B strongly and specifically inhibited astacin and meprin type metalloproteinases belonging to the metzincin superfamily, which also comprises MMPs, ADAMs, ADAMTS and other zinc proteinases^44,45^.

Fetuin-A has been identified as an important carrier of calcium phosphate in the context of matrix mineralization^19^ and additional roles have been suggested^46,47^. Fetal bovine serum derived fetuin (now known to contain mainly fetuin-A) readily associated with and inhibited trypsin^48^. However, trypsin activity could be recovered by prolonged incubation and this recovery was impeded by the presence of calcium. Fetuin integrity was not studied at the time. We confirmed transient inhibition of trypsin by recombinant fetuin-A. More importantly, we determined that fetuin-A is readily cleaved by trypsin and chymotrypsin (see Fig. 3). We conclude that fetuin-A is a preferred substrate of serine proteinases and was mistaken for an inhibitor in this and similar studies, because fetuin-A is cleaved before synthetic substrate of the proteolysis assay, but the state of fetuin-A itself was not studied. Similarly, fetuin-A turned out to be also a substrate for astacin metalloproteinases (this report) and for matrix metalloproteinases^49^. This might have physiological implications, which are not understood in detail yet. For example, the cleavage of fetuin-A by matrix degrading enzymes could impair extracellular matrix mineralization.

Vertebrate fetuins capable of inhibiting zinc metalloproteinases had been discovered also in reptiles and fishes. For example the pit viper (*Bothrops jararaca*) plasma protein BJ46a was found to inhibit ADAM-like snake venom metalloproteinases^50^, and a carp (*Cyprinus carpio*) plasma-derived fetuin-like protein inhibited the immune defense-associated astacin-metalloproteinase nephrosin^51,52^. Sequence alignment suggests that fish fetuin is more similar to mammalian fetuin-A than to fetuin-B, yet our work suggests that is functionally more related to mammalian fetuin-B. We speculate that the functional divergence of fetuins, which is seen in mammals, has evolved later in vertebrate evolution. Further work is needed to clarify this point as altogether three fetuin-like transcripts are found in the e.g. zebrafish genome, two of which are fetuin-B like^15^.

The inhibition of the metalloproteinase ovastacin by fetuin-B was the first observation of an astacin-like zinc-proteinase being antagonized by a member of the cystatin protein superfamily in mammals^2^. Pro-ovastacin is proteolytically inactive and requires removal of an amino-terminal pro-peptide to gain activity. Additionally, there is trimming at the C-terminal end. This report provides evidence for the activation of pro-ovastacin by a proteinase with trypsin-like cleavage specificity. In mouse oocytes two forms of ovastacin were detected *in vivo*, the 44 kDa pro-ovastacin and the mature ovastacin of 29 kDa, which is produced intracellularly by a hitherto unidentified proteinase^4^. Recombinant 46 kDa pro-ovastacin expressed in insect cells was processed by plasmin *in vitro*, liberating a catalytically active 28 kDa fragment that was identified by N-terminal sequencing and mass spectrometry to contain positions 86LLSV–to–NCSR283 of the complete ovastacin sequence (see Fig. 1). This variant likely corresponds to the 29 kDa form observed in mouse oocytes. The additional 31 kDa form is only partially processed on the C-terminal side and carries an elongation of 26 residues as determined by mass spectrometry. Minor differences in molecular masses (46 kDa vs. 44 kDa or 29 kDa vs. 28 kDa) are probably due to alternative modifications in mouse compared to insect cells.

The observation that pro-ovastacin can be activated by trypsin-activated plasmin and even better by a combination of plasmin and the trypsin-like proteinase t-PA, suggest thats a finely tuned proteolytic network controls the fertility of oocytes. This proteolytic network likely involves trypsin-like serine proteinases, which mediate ovastacin processing, active ovastacin, which cleaves the zona pellucida protein ZP2 thereby mediating definitive zona pellucida hardening, and fetuin-B, which regulates ovastacin activity^2,4,53,54^. Trypsin-like proteinases such as the tissue-type plasminogen activator (t-PA) exist in oocyte cortical granules^40^, and plasminogen exists in the follicular and oviductal fluids^41,42^. Both proteinases could be involved in a step-wise activation of pro-ovastacin. The observation that t-PA, which is secreted from oocytes^40^, on its own was not capable of activating ovastacin, whereas the combination of t-PA and plasminogen proved more effective than trypsin-activated plasmin alone, suggests that the partial intracellular activation of pro-ovastacin^4^ is mediated by a hitherto unidentified tryptic activity possibly present in cortical granules. Candidate serine proteinases were identified by proteomic analysis of the oocyte secretome^55^.

Changing expression systems did not alter inhibitory potency of fetuin-B. Bacterially expressed fish fetuin, truncated after the two cystatin-like domains, has inhibitory potency against astacin proteinases. This indicated that the presence of the C-terminal domain, glycosylation or other eukaryotic posttranslational modifications did not contribute essentially to function. This is in line with unchanged functional properties in fetuins expressed in High five insect cells, and mammalian COS-7 and CHO cells, respectively. Collectively, these results suggest that the cystatin-like protein domains are necessary and sufficient for the inhibitory function of fetuin-B.

Mouse ovastacin, human meprin α and β, zebrafish nephrosin and crayfish astacin are effectively inhibited by mouse fetuin-B. The only astacin proteinases resistant to fetuin-B inhibition were the members of the BMP-1/tolloid subfamily. As shown by crystal structure analysis^56^ and mass spectrometry^57^, these enzymes have in common a unique vicinal disulfide bond located in the substrate binding β-strand in intimate distance to the zinc ion of the active site. This arrangement most likely prevents access of fetuin-B to the active site.

Mouse fetuin-A did not show inhibitory activity against any of the enzymes tested. Hence, the inhibitory effect seems restricted to fetuin-B only. This seemingly contradicts our previous statement that fetuin-A can inhibit meprin proteinases^43^. We know now that commercial fetuin preparations contain mostly fetuin-A, but also sufficient fetuin-B to explain the observed inhibition given the strong inhibitory activity of fetuin-B^2^. Consequently, commercial antibodies against (contaminated) fetuin-A inevitably cross-reacted with fetuin-B and neutralized both proteins as observed^53^.

Interestingly, fetuin-A may influence metalloproteinase activity nevertheless explaining reports of fetuin-MMP interaction. In the present study we noticed an enhancing/stabilizing effect of fetuin-A in its interaction with ovastacin (see Supplementary Fig. 2a; and ref.^2^) and also for meprin α and β (data not shown). Similarly, protease stabilization by fetuin was reported for other metzincin proteinases such as pro-MMP-3 and (pro-) MMP-9^58-60^. Together, these observations suggest a supporting, scaffolding role for fetuin-A.

Fetuin-A and fetuin-B do not show any inhibitory effect against other metzincins or proteinases of other mechanistic classes. Regarding mammalian astacins, the inhibitory effect of fetuin-B was only observed against ovastacin and meprins. Beyond, other targets were fish nephrosin and the prototypical crayfish astacin. However, other metzincin metalloproteinases such as MMP-2, MMP-9, MMP-13, ADAM10 are most likely not physiological targets of fetuin-B, since they were not inhibited. Other proteinases not antagonized by fetuins are cysteine cathepsins-B, -K, -S and papain, the aspartate proteinase cathepsins-D, serine proteinases like trypsin, chymotrypsin or plasmin. A special case is the caspase-like cysteine proteinase legumain, which has been shown to be inhibited by cystatin-C-like members of the cystatin superfamily^14^, but – surprisingly – not with the N-terminus and two hairpin-loops as in the classic interaction with papain-like cysteine proteinases, but rather with a unique legumain-specific reactive site observed biochemically earlier by Abrahamson and coworkers^61^. We checked legumain for inhibition by fetuin-B and did not observe any effect, which is consistent with the absence of the typical asparaginyl-loop exposed by cystatin-C and -D for inhibition of legumain-like enzymes (Table 1).

In conclusion, fetuin-B emerges as a novel mammalian plasma proteinase inhibitor, selectively targeting members of the astacin-family of zinc metalloproteinases, namely ovastacin, meprin α and meprin β. While the regulated inhibition of ovastacin by fetuin-B has been shown to be essential and indispensable for female fertility, the consequences of meprin inhibition are less well understood, but could potentially affect many physiological functions. Meprins are pivotal in proteolytic networks controlling angiogenesis, immune defense, extracellular matrix assembly and general cell signaling, which might implicate consequences for inflammation, fibrosis, neurodegenerative disorders and cancer^25,27–33,36–38^.

## Methods

Enzymes and substrates were prepared as described below or obtained from commercial sources as listed in Table 2.

**Table 2:** Enzymes and substrates.

Enzyme	Substrate	Ex/Em [nm]	Turnover
***Serine proteinases***			
trypsin^a^	Boc-FSR-Amc^b^	380/460	trypsin^a^
plasmin^c^			
t-PA^d^			
chymotrypsin^e^	AAF-Amc^b^	380/460	chymotrypsin
***Cysteine proteinases***			
papain^f^	Boc-FSR-Amc^b^	380/460	trypsin^a^
cathepsin B^g^			
cathepsin K^g^			
cathepsin S^g^			
legumain^h^	Z-AAN-Amc^b^	380/460	legumain
***Aspartate proteinases***			
cathepsin D^g^	Mca-GKPILFFRLK(Dnp)-dR-NH_2_^g^	328/405	proteinase K^f^
***Metalloproteinases***			
MMP-2^g^	Mca-PLA-Nva-Dap(Dnp)-AR-NH_2_^h^	320/405	proteinase K^f^
MMP-8^h^			
MMP-9			
MMP-13^h^	Mca-PLGL-Dap(Dnp)-AR-NH_2_^h^	320/405	proteinase K^f^
ADAM10^h^	Mca-PLAQAV-Dpa(Dnp)-RSSSR-NH_2_^h^	320/405	proteinase K^f^
TLL-2	Ac-RE(Edans)-DR-Nle-VGDDPY-K(Dabcyl)-NH_2_^i^	350/520	proteinase K^f^
BMP-1			
ovastacin			
meprin α			
meprin β			
astacin	Dns-PKRAPWV^i^	280/350	proteinase K^f^
	Mca-GSPAFLA-K(Dnp)-dR-NH_2_^g^	320/405	proteinase K^f^
nephrosin	Mca-PLAQAV-Dpa(Dnp)-RSSSR-NH_2_^h^	320/405	proteinase K^f^

^a^Merck, Darmstadt, Germany; ^b^Bachem, Bubendorf, Switzerland; ^c^ABCAM Cambridge, UK; ^d^Hematologic Technologies, Vermont, USA; ^e^Serva, Heidelberg, Germany; ^f^Sigma-Aldrich, Taufkirchen, Germany; ^g^Enzo Life Sciences, Lörrach, Germany; ^h^R&D Systems Europe Ltd, Oxon, UK; ^i^Biosyntan, Berlin, Germany; Ac: acetyl; ADAM: A Disintegrin and Metalloproteinase; Amc: 7-amido-4-methylcoumarin; BMP: bone morphogenetic protein; Boc: t-butyl-oxycarbonyl; Ex: excitation wave length; Em: emission wave length; Mca: 7-methoxycoumarin-4-yl-acetyl; Dnp: 2,4-dinitrophenyl; Dap: L-2,3-diaminopropionyl; Dap(Dnp): L-2,3-diaminopropionyl-N3-2,4-dinitrophenyl; EDANS: 5-((2-aminomethyl)amino)naphtalene-1-sulfonic acid; Dans: N,N’-dimethylamino-naphtalene-1-sulfonic acid; MMP: matrix metalloproteinase; Na: nitroanilide; Nva: norvaline; Dabcyl: N,N’-diamino-phenyl-azo-4-benzoic-acid; Suc: succinyl; t-PA: tissue-type plasminogen activator; Z: benzyloxycarbonyl. ‘Turnover’ indicates the enzyme used (at 20 μg/1 μl) for complete substrate turnover after initial rate determination.

### Recombinant Enzymes

Recombinant mouse pro-ovastacin (Q6HA09)^2^, human meprin α (Q16819)^62^, human meprin β (Q16820)^63^, hBMP-1 (P13497)^64^, *Astacus astacus* astacin (P07584)^65^ and human MMP-9 (P14780)^66^ (a kind gift of Ghislain Opdenakker, Leuwen, Belgium) were prepared as described. For proteolytic activation, pro-ovastacin was mixed with human or murine plasmin in a molar ratio of 10:1 in 50 mM Tris/HCl pH 7.4, 150 mM NaCl buffer and incubated for 30 min at 37 °C before addition of 10 mM 4-(2-aminoethyl) benzenesulfonyl fluoride hydrochloride (AEBSF/Pefabloc^**®**^, Sigma-Aldrich, Taufkirchen, Germany) and incubation for another 10 min. Under the same conditions pro-ovastacin was activated with trypsin (300:1), t-PA (6:1) or a combination of plasminogen and t-PA (10:1:1). Activation was verified in an activity assay using fluorogenic substrates (Table 2). The activation by plasmin was confirmed by casein zymography performed under non-reducing conditions. The plasmid encoding the proteinase domain of human TLL-2 (Q9Y6L7), TLL-2 cat was a gift of the Novartis Institute for Biomedical Research (Basel, Switzerland). TLL-2 was expressed in *E. coli*^56^ renatured from inclusion bodies^67^, purified by gelfiltration and dialyzed against 50 mM Hepes, pH 7.4, 150 mM NaCl, 5 mM CaCl_2_, 0.02% octyl-β-D-glucopyranoside for further use. Recombinant C-terminally Strep-tagged *Danio rerio* nephrosin (Gene ID: 404039) was expressed in insect cells using the protocol developed for meprin^62^^,^^63^. Nephrosin was concentrated by ammonium sulfate precipitation and purified via Strep-tactin affinity chromatography as published for meprin α^62,63^.

### Fetuins

Bovine fetuin (bF) and bovine asialofetuin (bF asialo) from fetal plasma were obtained from Sigma-Aldrich (Taufkirchen, Germany). Mouse fetuin-A (P29699) and mouse fetuin-B (Q9QXC1) were cloned in pFASTBac 1 vectors and expressed in baculovirus transduced High Five cells as published for meprin α^62,63^, or cloned in AdEasy vectors and expressed in adenovirus transduced COS-7 cells, or cloned in pcDNA^TM^ 3.4 TOPO vectors (Thermo Fisher Scientific, Waltham, USA) and expressed in plasmid transfected CHO cells. Recombinant fetuin production in COS-7 cells was performed as described previously^2^. Protein expression in CHO Lec 3.2.8.1 cells and ExpiCHO-S cells (Chinese dwarf hamster ovary cells; Thermo Fisher Scientific, Waltham, USA) was performed according to the manufacturer’s specifications. Twenty hours after transfection 1 µg/mL tunicamycin was added to the medium. For purification the proteins were affinity purified on Ni-NTA-affinity matrix equilibrated with 25 mM imidazole, 200 mM NaCl, 10 mM Tris-HCl pH 7.4. The imidazole concentration was increased to 50 mM to remove unspecific bound proteins and to 100 mM for elution of the target protein. Imidazole was removed afterwards by dialysis or gel filtration. The purity of the fetuin proteins was judged by SDS-PAGE followed by Coomassie brilliant blue staining and Western blot. The two cystatin like domains of fish (*Cyprinus carpio*) fetuin-A (Q801ZP)^51^ corresponding to amino acid sequence positions 21 to 282 were cloned into the pQE-TriSystem His∙Strep 2 Vector (Qiagen, Hilden, Germany), which provides flanking sequences encoding a 5′-Strep-tag and a 3′-8xHis-tag. Expression in bacteria (*Escherichia coli*), purification of inclusion bodies, renaturation and affinity purification of fish fetuin-A followed an elaborated protocol^67^.

### Electrophoresis

SDS-PAGE was set up according to ref.^68^. For zymography, 0.1% casein (Hammarsten grade, Sigma-Aldrich, Taufkirchen, Germany) was added to the separation gel. All other buffers were prepared as described^69^. Proteins in sample buffer (sample loading buffer: 42 mM ammediol/HCl pH 8.37, 1% (w/v) SDS, 0.01% (w/v) NaN_3_, 20% (w/v) glycerol, 0.05% (w/v) bromophenol blue were applied onto a 3.8% stacking gel and separated in a 7.5% separation gel at 80 V (running buffer: 25 mM Tris, 192 mM glycine, pH 8.3, 0.02% (w/v) SDS). After electrophoresis the gels were incubated 3 * 20 min in washing buffer (50 mM Tris/ HCl pH 7.5, 200 mM NaCl, 5 mM CaCl_2_, 0.02% (w/v) NaN_3_, 5 µM ZnCl_2_, 2.5% (w/v) Triton X-100) on a shaker and at 37 °C for 48 h in renaturing buffer (washing buffer without Triton). Staining occurred for 30 min in 0.125% Coomassie R-250, 50% ethanol, 20% acetic acid in ultrapure water, destaining was achieved in 30% ethanol, 1% acetic acid in ultrapure water.

### Western blotting and glycoanalysis

For immunoblotting the separated proteins were transferred to polyvinylidene difluoride (PVDF)-membrane (Immobilon^®^FL, MerckMillipore, Darmstadt, Germany) via semi-dry blotting^70^ at constant 20 V for 30–60 min. Immunodetection of fetuin fragments was performed with rabbit anti-mouse fetuin-A and rabbit anti-mouse fetuin-B antibodies^2^ or with anti-His-tag antibodies (Qiagen, Hilden, Germany; 1:2000 in 1% BSA TBS-T). For glycoanalysis proteins were blotted onto a nitrocellulose membrane (Sigma-Aldrich, Taufkirchen, Germany, Germany) using a semi-dry blotting device (BioRad, München, Germany). The membrane was blocked with 2,5% BSA powder in PBS-T (PBS supplemented with 0.05% Tween-20, Applichem, Darmstadt, Germany) for 1 h at 37 °C. Blotted proteins were probed with lectins (Vector Laboratories, Burlingame, USA) from *Sambucus nigra* (EBL,), *Erythrina cristagalli* (ECL), *Griffonia simplicifolia* (GS-II) for terminal sialic acid, galactose and N-Acetylglucosamin respectively. Lectins were diluted 1:2000 in PBS-T and applied for 1 h at 37 °C. Streptavidin-POD conjugate (Streptavidin covalently coupled to horseradish peroxidase, Sigma) was diluted 1:5000 in PBS-T and incubated for 1 h at 37 °C. Afterwards, membranes were washed three times with PBS-T for 5 min. Conjugate bound to lectins was detected by chemiluminescence in substrate solution (0.1 M Tris/HCl, pH 8.5, 1.25 mM 3-aminopthalhydrazide, 0.45 mM p-coumaric acid, 0.015% hydrogen peroxide) using the fluorescence imager Fuji LAS Mini 4000 (GE Healthcare, Freiburg, Germany).

### Enzymatic assays and determination of kinetic constants

Kinetics of substrate hydrolysis was monitored in a temperature-controlled Varioskan Flash 3001 spectral plate reader equipped with the SkanIt Software 2.4.3.RE (Thermo Scientific, Dreieich, Germany) in a total volume of 100 µl at 37 °C. The reactions were started by addition of substrate and initial velocities were recorded for at least 360 s (>70 times for 100 ms at intervals of 5 s). Thereafter, in the case of quenched fluorescent substrates, 20 µg of concentrated proteinase in 1 µl buffer to cleave all remaining substrate, and the reaction was monitored for another 15 min. For calculation of the rate of substrate turnover the following formula was used: v = [S]*m/ΔF, m [F/t]: slope of initial substrate turnover; ∆F: maximum fluorescence intensity after complete substrate turnover, [S]: substrate concentration. Enzymes and substrates are listed in Table 2. Kinetic parameters were analyzed using GraFit 4 (Erithacus Software, Wilmington House, UK); K_i_ values were calculated using the equation published by Joseph Bieth^71^.

In activity or inhibition assays ovastacin was used at concentrations in the range of 0.018–5 µM. Fetuin-A and fetuin-B were tested at concentrations of 0.4 nM–1.2 µM. Enzyme activity measurements were started by addition of 10–32 µM Ac-RE(Edans)-DR-Nle-VGDDPY-K(Dabcyl)-NH_2_ dissolved in 0.4% DMSO (dimethyl sulfoxide).

Crayfish astacin^65^ was assayed after pre-incubation for 10 minutes at 37 °C with fetuin-A (0.25 nM–2 µM, 10 nM enzyme) or fetuin- B (0.2 nM–0.9 µM, 1 nM enzyme), before addition 14 µM Mca-GSPAFLA-K(Dnp)-dR-NH_2_ in 0.5% DMSO or 0.4 mM Dns-PKRAPWV substrate both in 50 mM Hepes pH 8.0.

TLL-2 (180 or 250 nM) and BMP-1 (10 nM) were pre-incubated for 10 min at 37 °C with fetuin-A or fetuin-B (0.005 nM–3 µM). The reaction in 50 mM Hepes, pH 7.2, 150 mM NaCl, 5 mM CaCl_2_, 0.04% octyl-beta-D-glucopyranoside and 0.3% DMSO was started by addition of 20 µM or 24 µM Ac-RE(Edans)-DR-Nle-VGDDPY-K(Dabcyl)-NH_2_ substrate.

Pro-meprin α and pro-meprin β were activated by trypsin (70:1) for 45 minutes at 37 °C. Trypsin was inactivated by adding 10 mM Pefabloc^**®**^ (Sigma-Aldrich, Taufkirchen, Germany). 0.25 nM of each activated enzyme (50 mM Hepes pH 7.5) were pre-incubated for 10 min with fetuin-A or -B (0.22 nM–1 µM, 15 µl in 50 mM Hepes, pH 7.4, 150 mM NaCl, 2.5 mM CaCl_2_, 0.04% octyl-beta-D-glucopyranoside). The enzyme activity measurement was started by addition of 11, 20 or 32 µM Ac-RE(Edans)-DR-Nle-VGDDPY-K(Dabcyl)-NH_2_ in 0.4% DMSO.

10 nM nephrosin was pre-incubated with fetuin-A (10 nM–1 µM) or fetuin-B (0.02 nM–6.9 nM) for 10 min before addition of substrate (Mca-PLAQAV-Dpa(Dnp)-RSSSR-NH_2,_ 10 µM if fetuin-A, 12 µM if fetuin-B). The measurement with fetuin-A was run in 20.5 mM Tris/HCl pH 9.0, 135 mM NaCl, 0.25 µM ZnCl_2_, 0.0005% Brij^®^-35, 0.25% DMSO. For fetuin-B a buffer containing 22.25 mM Tris/HCl pH 9.0, 82.5 mM NaCl, 1.12 µM ZnCl_2_, 0.002% Brij^®^-35, 0.3% DMSO was used.

Human ADAM10 (19 nM) was pre-incubated for 10 min with 10 µl fetuin-B (300 nM or 600 nM) or with 10 µl fetuin-A (325 nM or 650 nM) in 25 mM Tris/HCl pH 9.0, 2.5 µM ZnCl_2_, 0.005% Brij^®^-35. By addition of 12 µM substrate Mca-PLAQAV-Dpa(Dnp)-RSSSR-NH_2_ 0.3% DMSO was introduced into the reaction buffer. Alternatively 38 nM enzyme were pre-incubated for 10 or 30 min with 1 µM fetuin-A or B and assayed with 20 µM substrate (0.5% DMSO).

Pro-MMP-2, pro-MMP-8, pro-MMP-9 or pro-MMP-13 were activated with 2 mM APMA (p-aminophenyl-mercuric acetate) at 37 °C for 2 h in 50 mM Tris/HCl pH 7.4, 200 mM NaCl, 5 mM CaCl_2_, 1 µM ZnCl_2_, 0.05% Brij^®^-35 (50 mM Tris/HCl pH 7.5, 150 mM NaCl, 10 mM CaCl_2_ and 0.05% Brij-35 in case of MMP-13). Activated enzymes (MMP-2 and MMP-9 20 nM, MMP-8 25 nM and MMP-13 3.9 nM) were pre-incubated for 10 min at 37 °C with fetuin-A or -B (20 nM -2 µM) The reactions were started by addition of 8 µM Dnp-PLGLWA-dR-NH_2_ (MMP-2, MMP-8 and MMP-9, 2.5% DMSO) or 20 µM Mca-PLGL-Dap(Dnp)-AR-NH_2_ (MMP-13, 0.7% DMSO) substrate.

50 nM human plasmin or human t-PA were pre-incubated for 10 min with 300 nM fetuin-B or 250 nM fetuin-A at 37 °C before the reaction was started by addition of 20 µM Boc-SFR-AMC substrate in 50 mM Hepes pH 7.4, 150 mM NaCl, 5 mM CaCl_2_, 0.02% octyl-β-D-glucopyranoside and 0.4% DMSO.

Bovine trypsin (5 nM) in 50 mM Hepes pH 7.5, 1% DMSO was pre-incubated with 20 µl fetuin-A and fetuin-B (0.1 µM or 1 µM in PBS) for 10 and 30 min at 37 °C, respectively, before starting the reaction by adding of 100 µM Boc-SFR-AMC substrate.

Bovine chymotrypsin (19 nM) was pre-incubated with 1 µM or 0.1 µM fetuin-A or -B for 10 minutes at 37 °C and assayed with 100 µM H-AAF-Amc^b^ substrate in 38 mM Tris-HCl, 53 mM CaCl2, pH 7.8, 0.2% DMSO.

Latent legumain was activated for 2 h in 50 mM NaOAC pH 4, 100 mM NaCl. 10 nM active legumain were pre-incubated with fetuin-A or fetuin-B (40 nM–400 nM) for 10 min before addition of 100 μM Z-AAN-AMC substrate. The measurement with fetuin-A was run in buffer containing 50 mM MES pH 5, 250 mM NaCl, 192 µM NaOAC, 22.5 mM Tris, 22.5% Glycerol, 1% DMSO.

150 nM papain in 50 mM Hepes pH 7,5 was pre-incubated with fetuin-A or fetuin-B (150 or 300 nM, 15 µl in 50 mM Hepes, pH 7.4, 150 mM NaCl, 2.5 mM CaCl_2_, 0.04% octyl-beta-D-glucopyranoside) for 10 min before addition of 50 µM Boc-SFR-AMC substrate in 0.5% DMSO.

140 nM cathepsin B was pre-incubated with fetuin-A or fetuin-B (10 nM–1 µM) for 10 min before addition of 50 µM Boc-SFR-AMC substrate. The measurement was run in 50 mM NaOAc pH 6, 1 mM EDTA, 0.5% DMSO.

Pro-cathepsin K (Table 2) was measured at 200 nM in a total volume of 100 µl. Activation occurs by incubation of 5 µl (8.5 µM) pro-cathepsin K with 1 µl activation buffer (32.5 mM NaOAc, pH 3.5) for 40 min at room temperature. Subsequently the enzyme were diluted to 1 µM by addition of assay buffer (50 mM NaOAc, 50 mM NaCl, 0.5 mM EDTA und 5 mM DTT, pH 5.5). 20 µl of this solution were combined with 20 µl fetuin-A or fetuin-B (to achieve final concentrations in the range of 0.1–1.0 µM in 100 µl test volume; both in cathepsin K assay buffer) and incubated for a further 10 min. The reaction was started with addition of the substrate Boc-SFR-AMC (Table 2) to an end concentration of 320 μM. A control contained 2.8 mM of the cysteine proteinase inhibitor E64.

50 nM cathepsin S were pre-incubated for 10 min with five different concentrations of fetuin A or B in a range of 0.05–1 µM each. Enzyme activity was measured in presence of 320 µM of the substrate Boc-SFR-AMC (Table 1).

40 nM cathepsin D (50 mM NaOAC pH4) was pre-incubated with fetuin-A or fetuin-B (10 nM or 1 µM, 10 µl 50 mM NaOAc, pH 6) for 10 min before addition of 30 µM Mca-GKPILFFRLK(Dnp)-dR-NH_2_ substrate (3.5% DMSO).

### Cleavage of fetuin-A and fetuin-B by trypsin, chymotrypsin, and plasmin

Fetuin-A (0.33 µM) or fetuin-B (0.33 µM) were incubated with trypsin (0.03 µM), chymotrypsin (0.03 µM) or plasmin (0.16 µM) for up to 60 minutes at 37 °C in 50 mM Hepes, pH 7.2, 150 mM NaCl, 5 mM CaCl_2_, 0.04% octyl-beta, D-glucopyranoside. The reaction was stopped by addition of electrophoresis sample buffer (end concentration 66.7 mM Tris, pH 6.8, 8.3% (v/v) glycerol, 0.08% (w/v) bromophenol blue, 3.3% (w/v) SDS, 40 mM DTT) and heated at 98 °C for five minutes prior to separation in a 12% SDS-PAGE under reducing or non-reducing conditions^68^.

### Cleavage of fetuin-A and fetuin-B by astacin metalloproteinases

Fetuin-A or fetuin-B (each 4 µl), 6 µM in 50 mM Hepes, pH 7.4, 150 mM NaCl, 5 mM CaCl_2_, 0.02% octyl-beta-D-glucopyranoside were treated at 37 °C for up to 24 h with the same volume of astacin (50 nM in 50 mM Hepes, pH 8.0), human meprin α (50 nM in 50 mM Hepes, pH 7.5), human meprin β (50 nM in 50 mM Hepes, pH 7.5), human BMP-1 and human TLL-2 (50 nM in 50 mM Hepes, pH 7.2, 150 mM NaCl, 5 mM CaCl_2_, 0.04% octyl-beta-D-glucopyranoside) and zebrafish nephrosin (50 nM in 20 mM Tris pH 8.0, 150 mM NaCl). The reactions were stopped by addition of boiling sample buffer and the proteins were separated in a 12% SDS gel before Western blot analysis.

### N-terminal sequencing and mass spectrometry

Human plasmin activated pro-ovastacin was submitted to SDS-PAGE and immunoblotting onto PVDF membrane. N-terminal sequence of the resulting 28 kDa band was determined via Edman-degradation^72^ by ChromaTec GmbH (Greifswald, Germany), who also identification other cleavage products, in gel bands by Maldi-TOF-TOF analysis.

## Supplementary information

Supplementary Figures and Information

## Data Availability

The datasets generated during and/or analysed during the current study are available from the corresponding author on reasonable request.
