# Supplementary material for: Mammalian plasma fetuin-B is a selective inhibitor of ovastacin and meprin metalloproteinases
Source: Sci Rep. 2019 Jan 24;9:546. doi: 10.1038/s41598-018-37024-5 (PMC6346019; doi:10.1038/s41598-018-37024-5)

Supplementary Figures and Information

Mammalian plasma fetuin-B is a selective inhibitor of ovastacin and meprin metalloproteinases

Konstantin Karmilin[1], Carlo Schmitz[2], Michael Kuske[1], Hagen Körschgen[1], Mario Olf[1], Katharina Meyer[1], André Hildebrand[1], Matthias Felten[1], Sven Fridrich[1], Irene Yiallouros[1], Christoph Becker-Pauly[3], Ralf Weiskirchen[4], Willi Jahnen-Dechent[2], Julia Floehr[2], and Walter Stöcker[1,*]

[1] Institute of Molecular Physiology, Cell and Matrix Biology, Johannes Gutenberg University Mainz, 55099 Mainz, Germany

[2] Helmholtz Institute for Biomedical Engineering, Biointerface Laboratory, RWTH Aachen University, Medical Faculty, 52074 Aachen, Germany

[3] Institute of Biochemistry, CAU, 24118 Kiel, Germany

[4] Institute of Molecular Pathobiochemistry, Experimental Gene Therapy and Clinical Chemistry RWTH, 52074 Aachen, Germany

* stoecker@uni-mainz.de

Important note:

This file contains **Supplementary Figures S1, S2 and S3** and original images of gels and blots therein, which explain the composition of the cropped images of the supplemental figures. **In addition there are original images of Figure 1 of the main manuscript**, which show the Ponceau S stained PVFD-membrane and the zymography gel. The zymography was difficult to photograph, due to the massive band produced by plasmin, which was used to activate pro-ovastacin. However, the band at the bottom of lane 7 of Fig. 1 is only observed in the presence of pro-ovastacin, not in the plasmin control (lane 5).

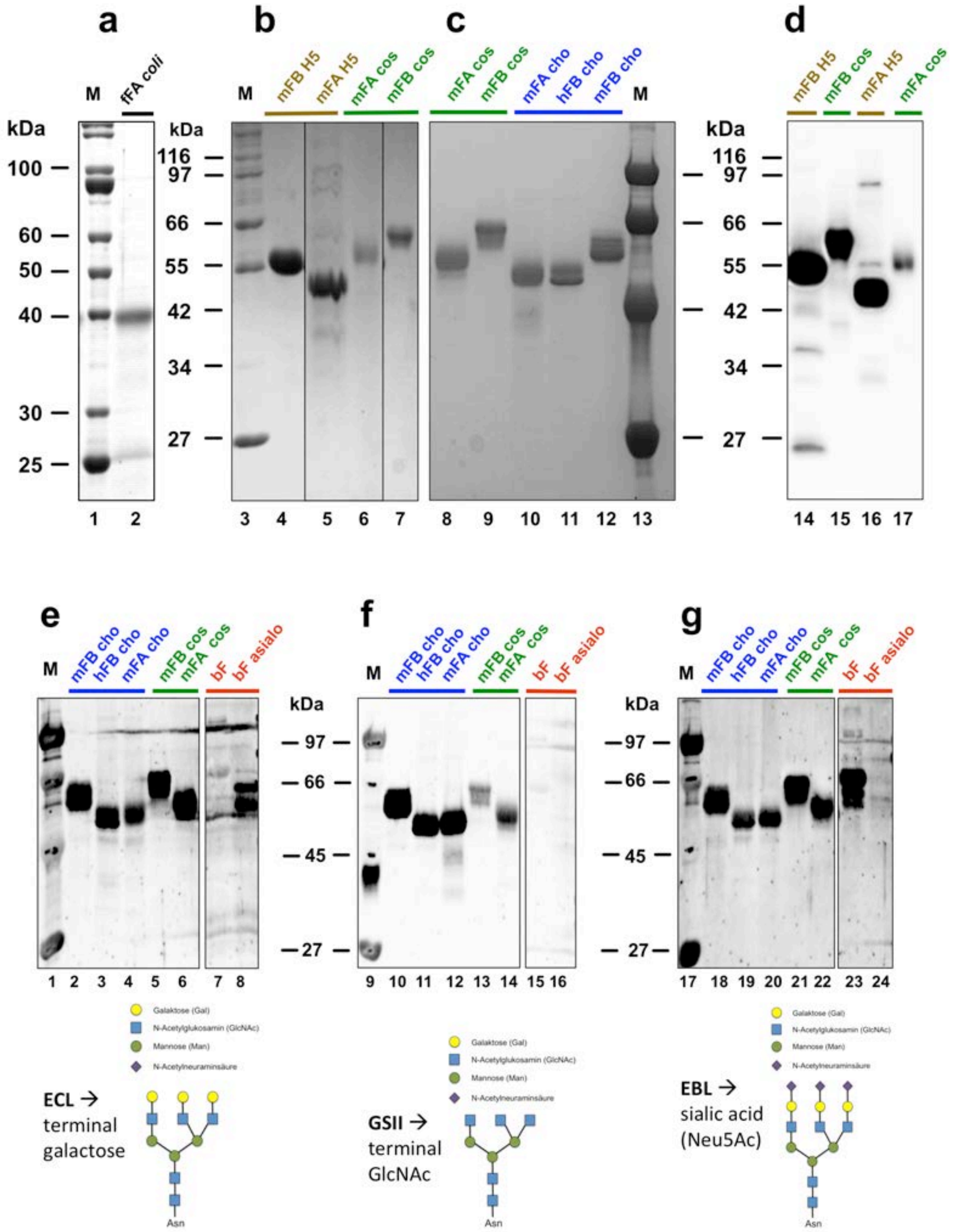

Supplementary Figure S1: Recombinant fetuin-A and fetuin-B variants from fish (fFA), human (hFB) and mouse (mFA, mFB) expressed in *Escherichia coli* (fFA *coli*), adenovirus transduced COS-7 cells (mFA cos, mFB cos, hFB cos), baculovirus infected High Five insect cells (mFA H5, mFB H5) and plasmid transfected Chinese hamster ovary cells (mFA cho, mFB cho, hFB cho)**. a-c,** Separation of 1-3 µg protein per lane in a 12% SDS-PAGE followed by Coomassie staining. The proteins from High Five insect cells had about 5 kDa lower mass due to truncated glycosylation; **d**, Western blot analysis using mouse anti-His-tag antibodies. **e-g** lectin-blots using biotinylated ECL-lectin (**e**) detecting terminal galactose, GS-II-lectin (**f**) detecting terminal N-acetyl-D-glucosamine (GlcNAc) and EBL-lectin (**g**), detecting terminal sialic acid. Terminally sialylated fetuin from fetal bovine serum (bF) and desialylated asialofetuin from fetal bovine serum (bF asialo) were used as controls.

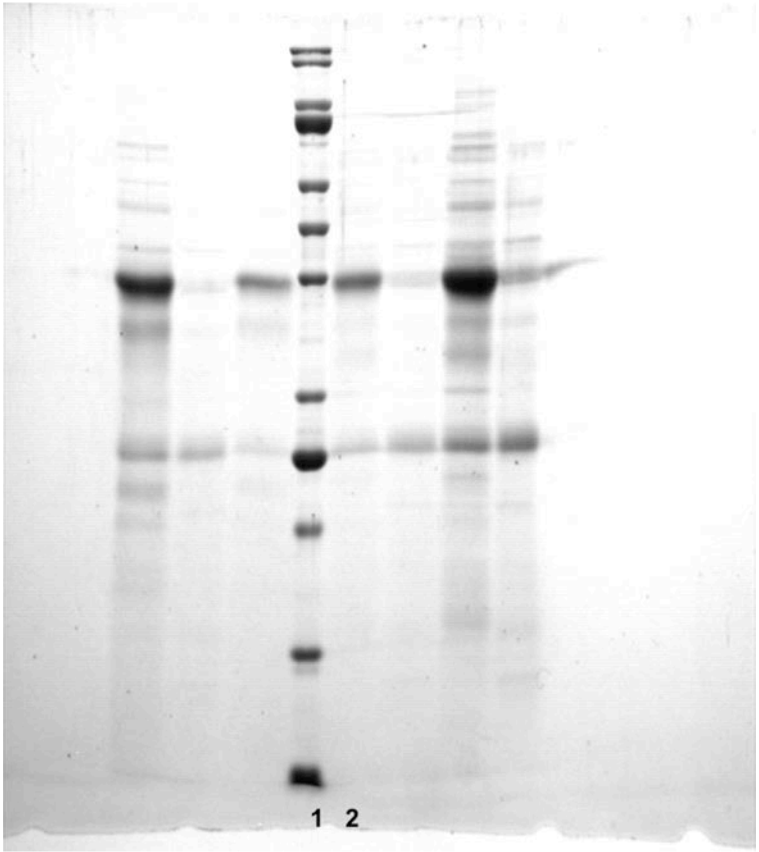

Supplementary Figure S1a original gel: The numbers indicate lanes corresponding to the cropped Supplementary Figure S1a.

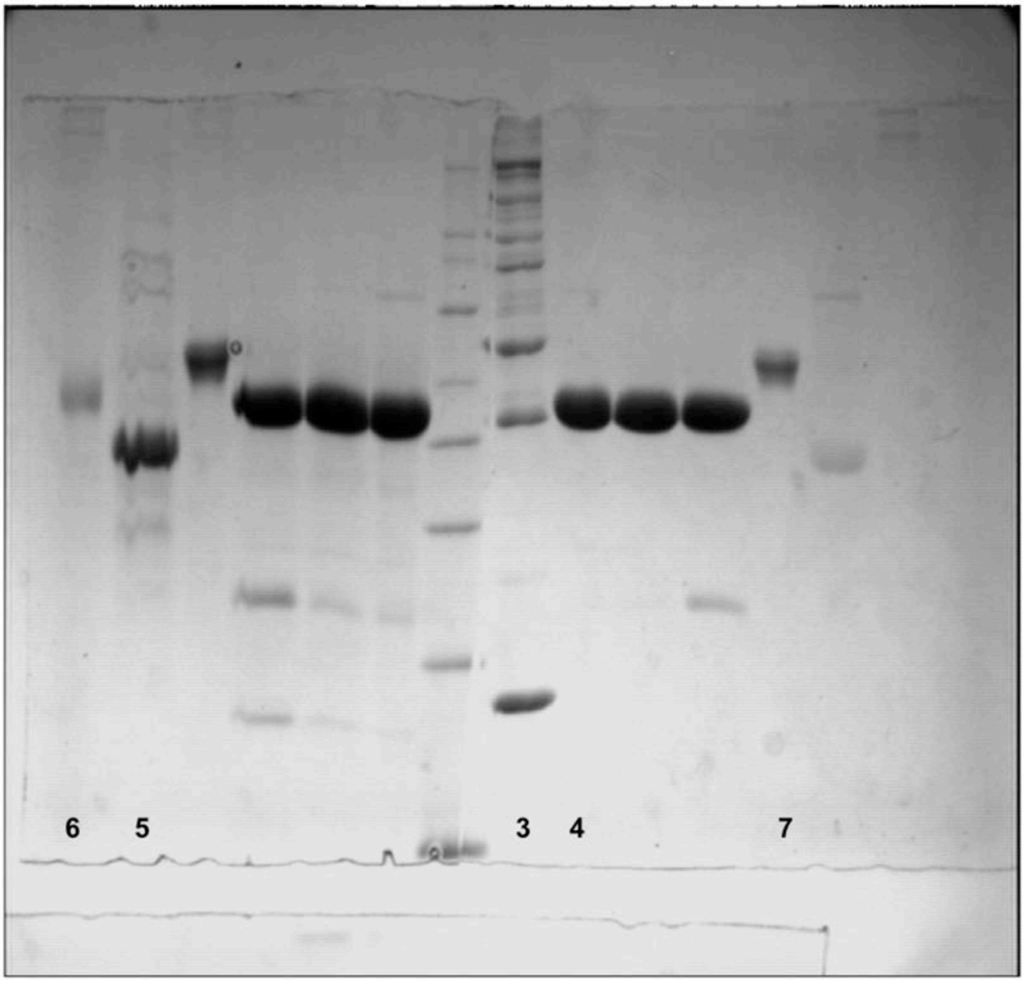

Supplementary Figure S1b original gel: The numbers indicate lanes corresponding to the cropped supplementary Figure S1b.

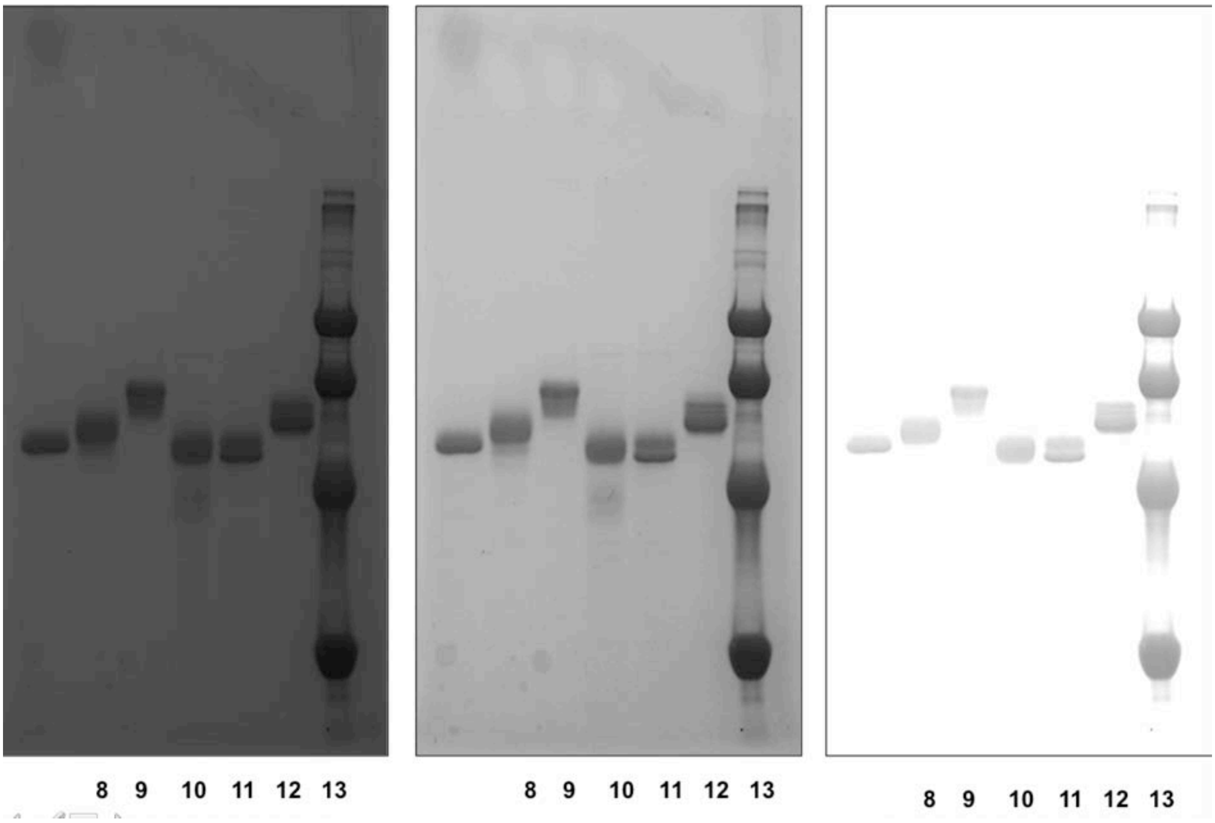

Supplementary Figure S1c original gel: The numbers indicate lanes corresponding to the Supplementary Figure 1c. The image in the middle was used in Supplementary Figure S1c. The images are displayed in triplicate with different adjustments of brightness and contrast. The picture in the middle was used in Supplementary Figure S1c.

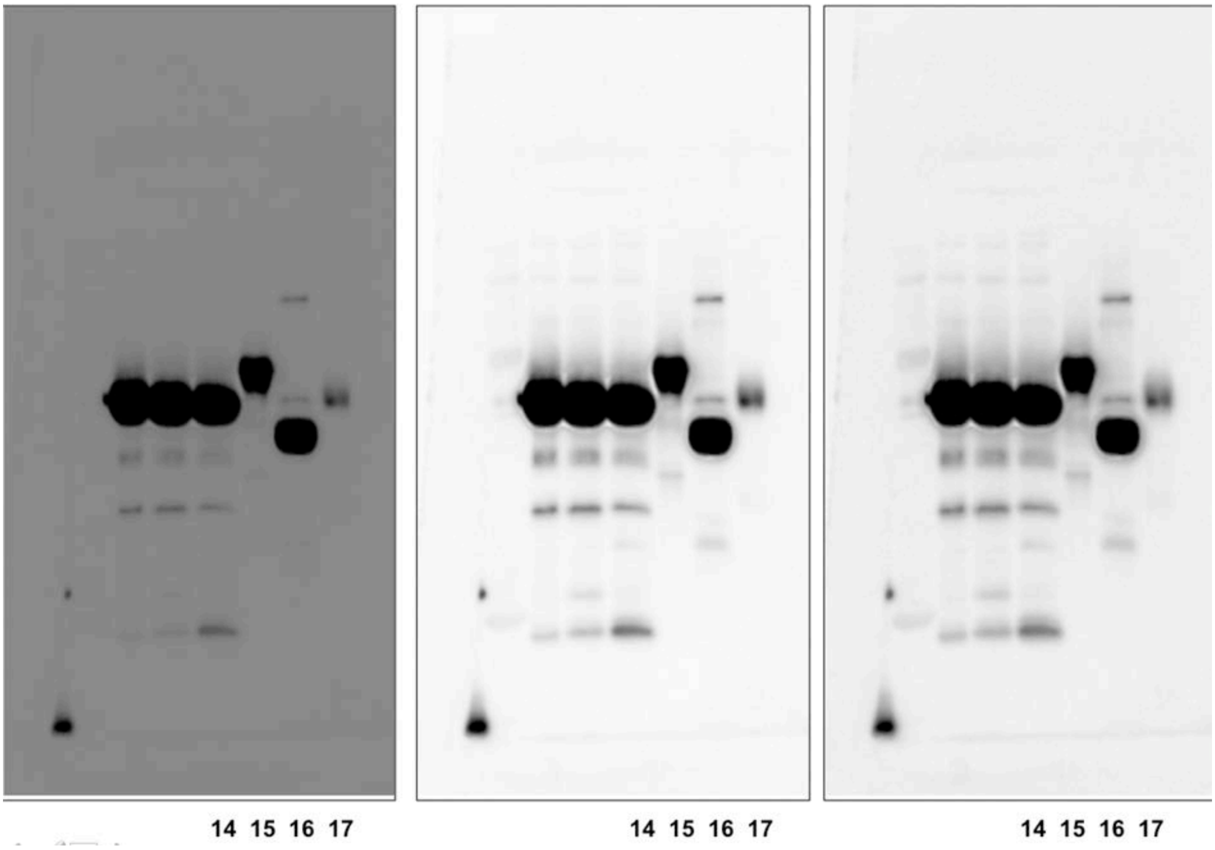

Supplementary Figure S1d original Western blot: The numbers indicate lanes corresponding to the cropped Supplementary Figure S1d. The images are displayed in triplicate with different adjustments of brightness and contrast. The picture in the middle was used in Supplementary Figure S1d.

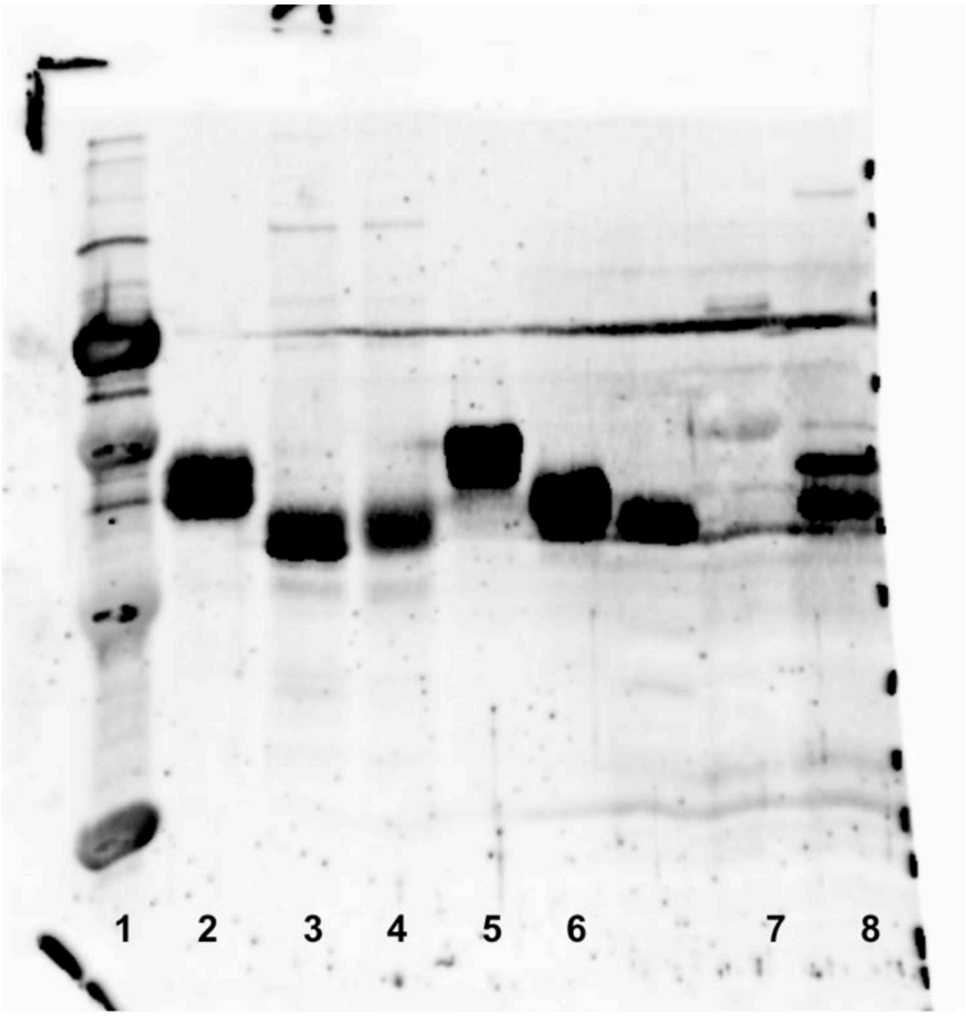

Supplementary Figure S1e original blot: The numbers indicate lanes corresponding to the cropped Supplementary Figure S1e.

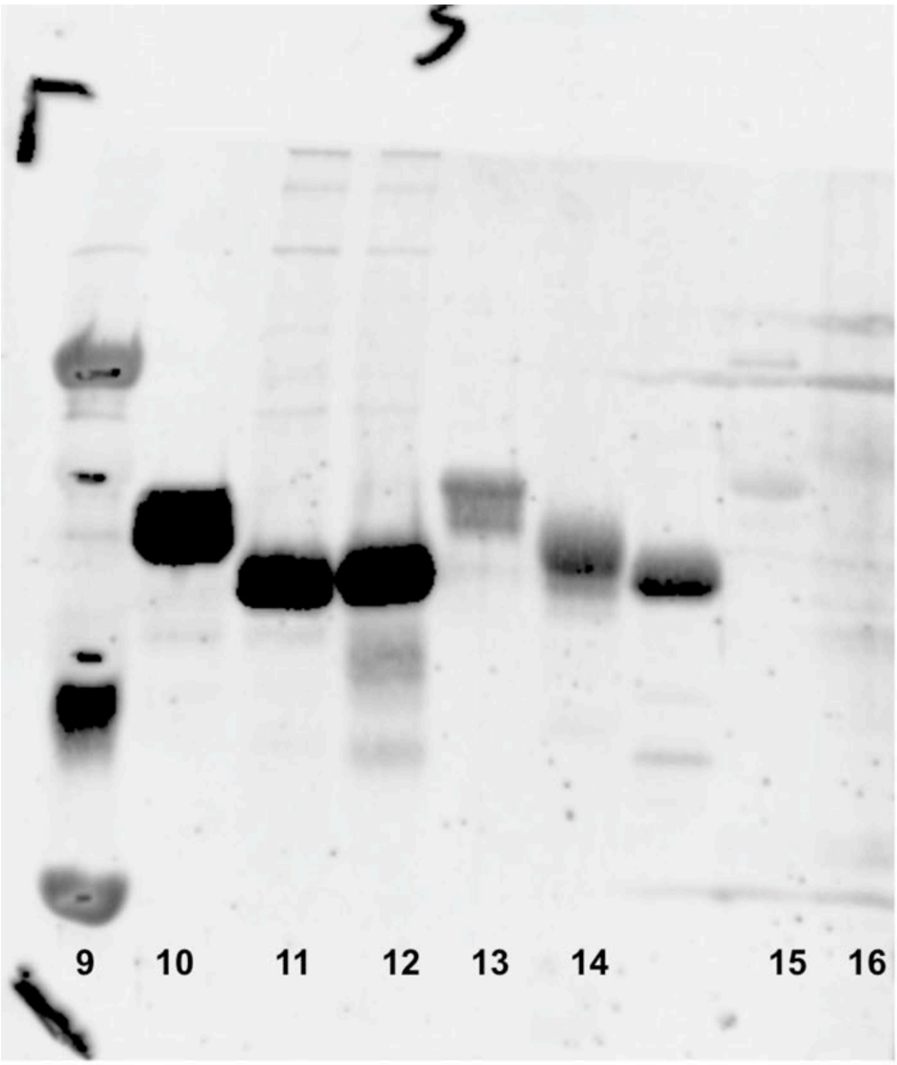

Supplementary Figure S1f original blot: The numbers indicate lanes corresponding to the cropped Supplementary Figure 1Sf.

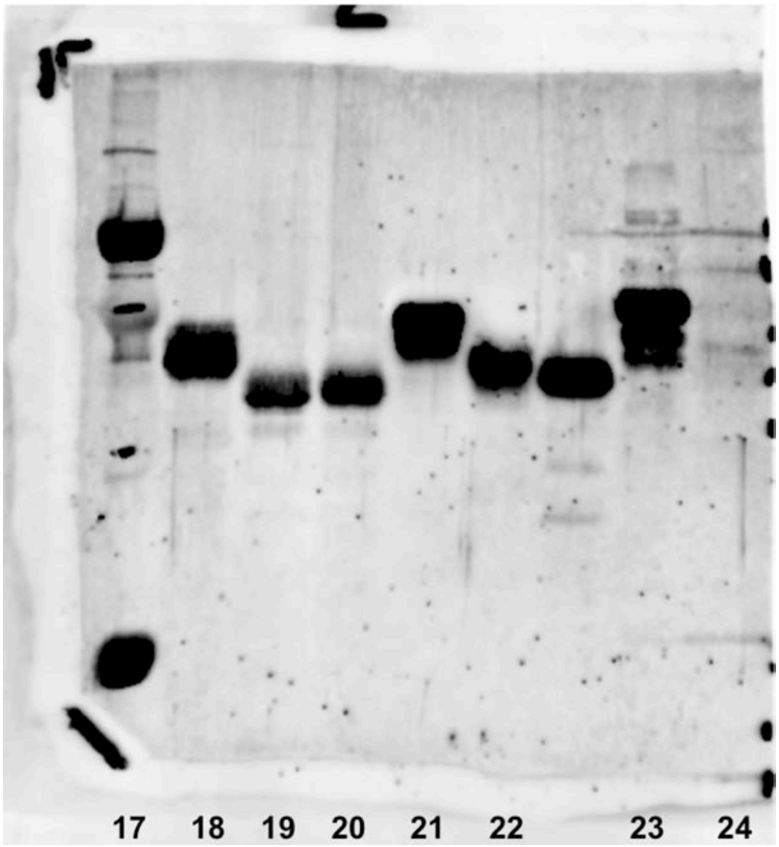

Supplementary Figure S1g original blot: The numbers indicate lanes corresponding to the cropped Supplementary Figure S1g.

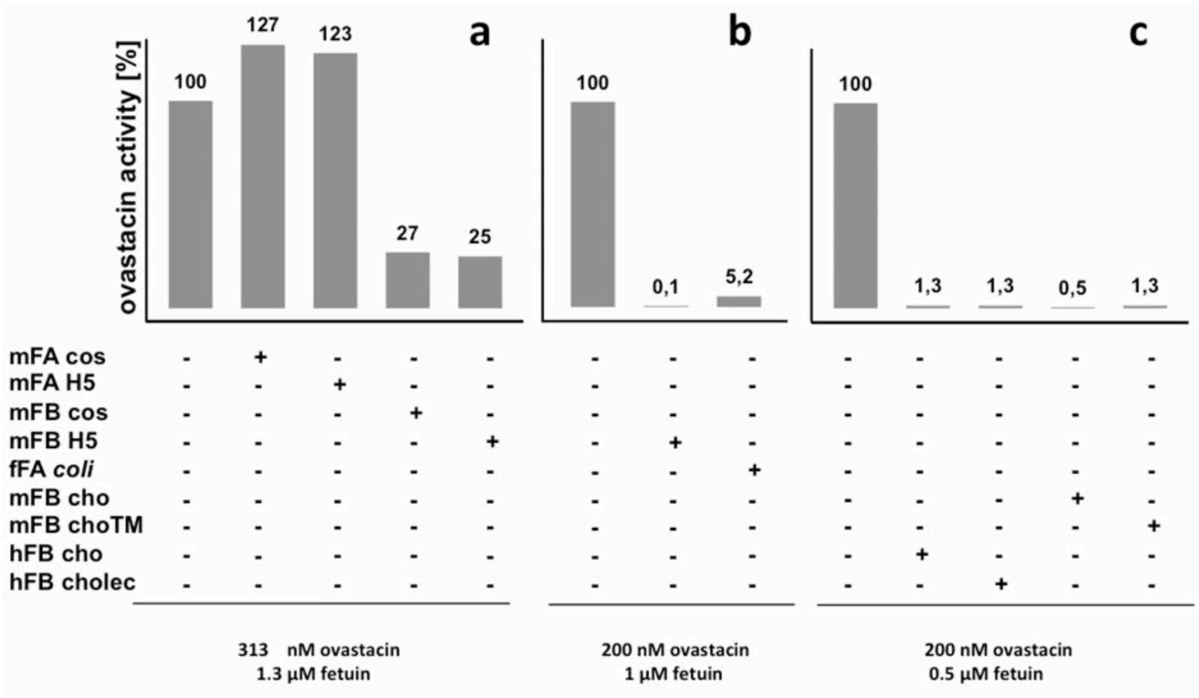

Supplementary Figure S2: Inhibition of ovastacin by recombinant fetuin variants expressed in plasmid transformed *Escherichia coli* (fFA *coli*), adenovirus transduced COS-7 cells (mFA cos, mFB cos, hFB cos), baculovirus transduced High Five insect cells (mFA H5, mFB H5) and plasmid transfected Chinese hamster ovary cells (mFB cho, mFB choTM, hFB cho, mFB cholec). mFB cholec was expressed in plasmid transfected mutant CHO lec1 cells, which are unable to perform complex and hybrid-type N-glycosylation; mFB choTM was expressed in presence of tunicamycin, inhibiting N-glycosylation. **a**, 100% activity of the ovastacin control corresponds to a substrate (Ac-RE(Edans)-DR-Nle-VGDDPY-K(Dabcyl)-NH_2) turnover rate of 3.6 nM/s measured in 50mM Tris/HCl pH7.4; **b**, 100 % of the ovastacin control corresponds to a substrate (Ac-RE(Edans)-DR-Nle-VGDDPY-K(Dabcyl)-NH_2) turnover of 6.87 nM/s measured in 50mM Tris/HCl, 150mM NaCl, 0,01 % Brij-35, pH7.4; **c,** 100 % of the ovastacin control corresponds to a substrate (Ac-RE(Edans)-DR-Nle-VGDDPY-K(Dabcyl)-NH_2) turnover of 5.86 nM/s measured in 50mM Tris/HCl, 150mM NaCl, 0,01 % Brij-35, pH7.4.

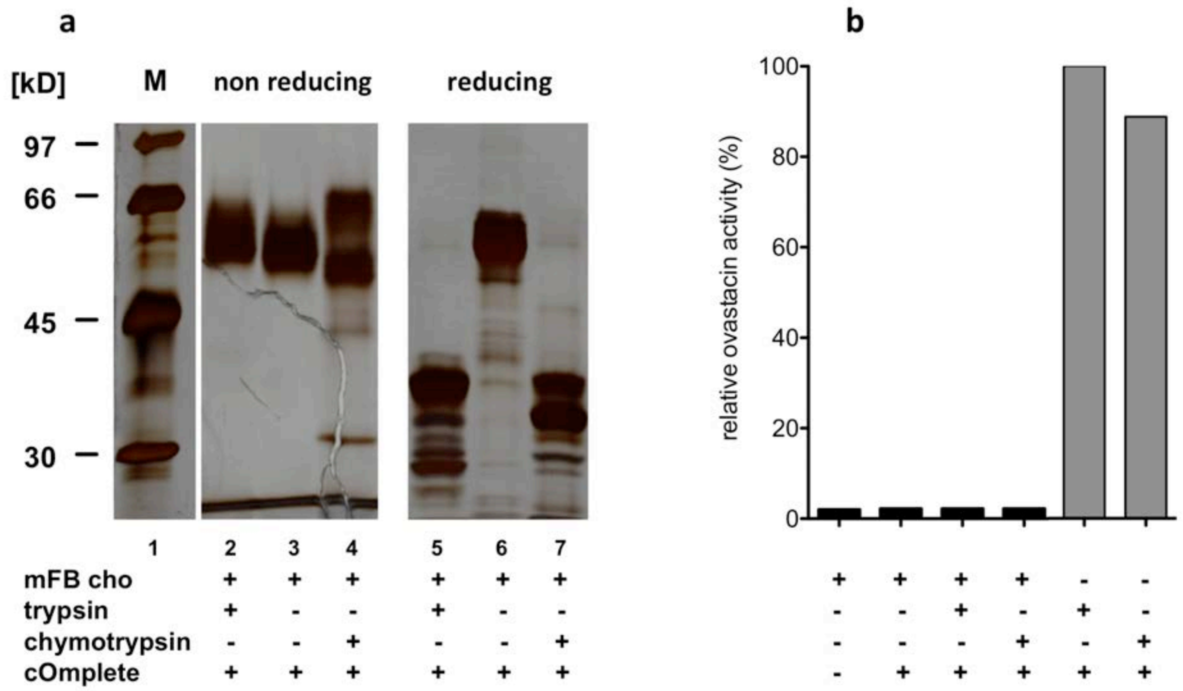

Supplementary Figure S3: Trypsin and chymotrypsin cleavage of fetuin-B do not abolish ovastacin inhibition. Trypsin and chymotrypsin were incubated with mFB cho for 10 minutes at 37°C. cOmplete^{TM} EDTA-free Protease Inhibitor Cocktail (Roche, Basel, Switzerland) was added to a final concentration of 80 mg/ml. **a,** Aliquots of the digestion mixture were analyzed by reducing and non-reducing SDS-PAGE and silver staining. Reducing SDS-PAGE revealed limited proteolysis of intact 60 kDa fetuin-B into fragments of 40 kDa and smaller. Non-reducing SDS-PAGE showed that the non-cleaved 60 kDa fetuin-B was left largely intact with a small upward shift of trypsin-cleaved fetuin-B to about 62 kDa, and the generation by chymotrypsin cleavage of 55 kDa and 65 kDa double bands. Major small cleavage fragments observed in reducing SDS-PAGE were absent in non-reducing SDS-PAGE suggesting disulfide bond-stabilization of fetuin-B cleavage fragments. **b**, trypsin and chymotrypsin-cleaved fetuin-B retained ovastacin inhibition.

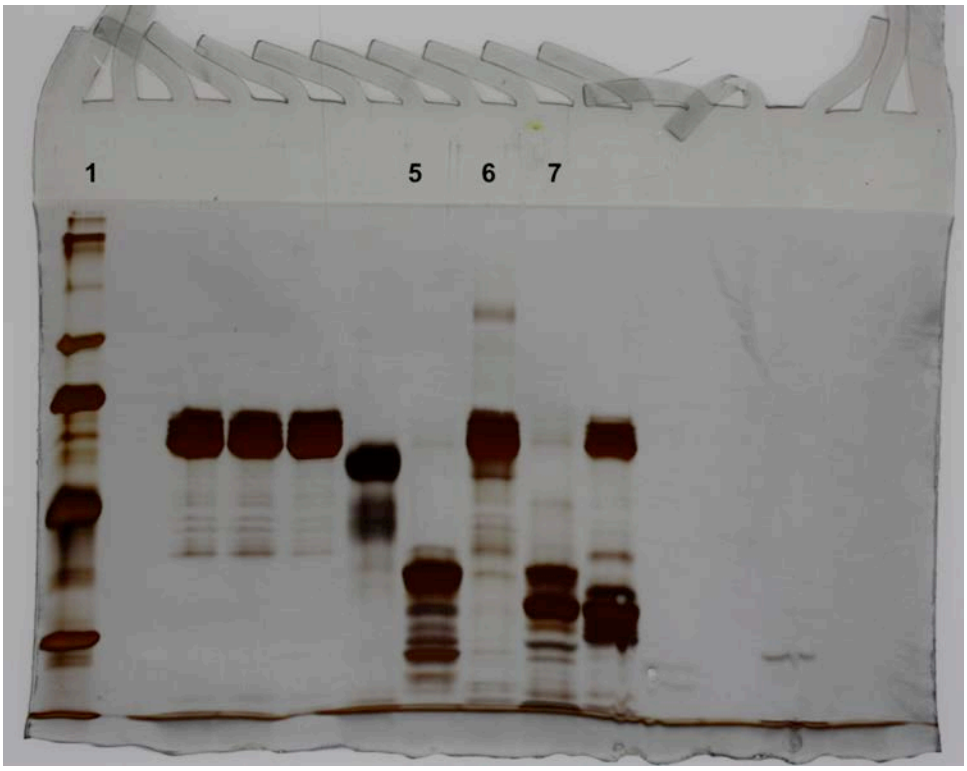

Supplementary Figure S3 original reducing gel: The numbers indicate lanes corresponding to the cropped Supplementary Figure S3.

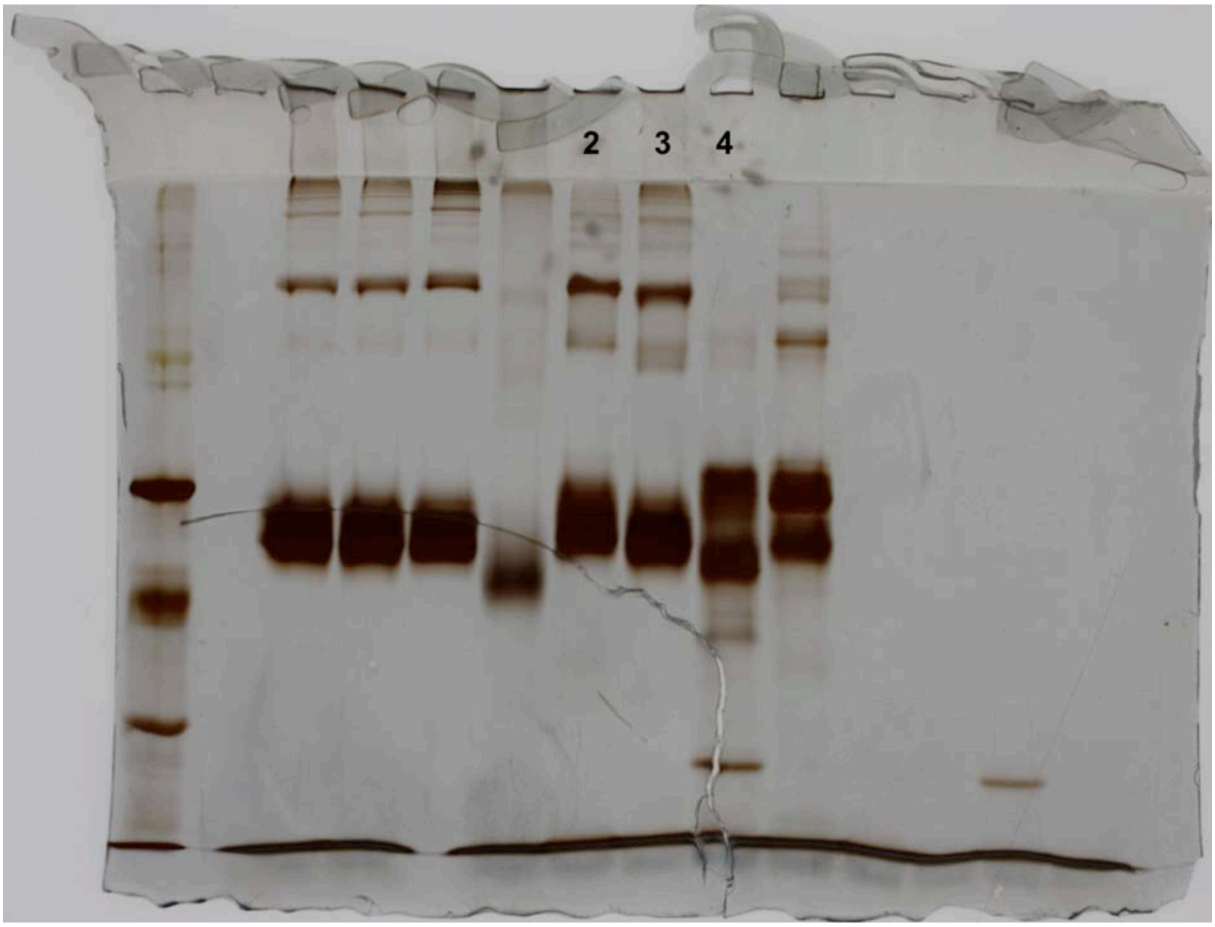

Supplementary Figure 3 original non-reducing gel: The numbers indicate lanes corresponding to the cropped Supplementary Figure 3.

Additional supplementary original images explaining Figure 1 of the main manuscript

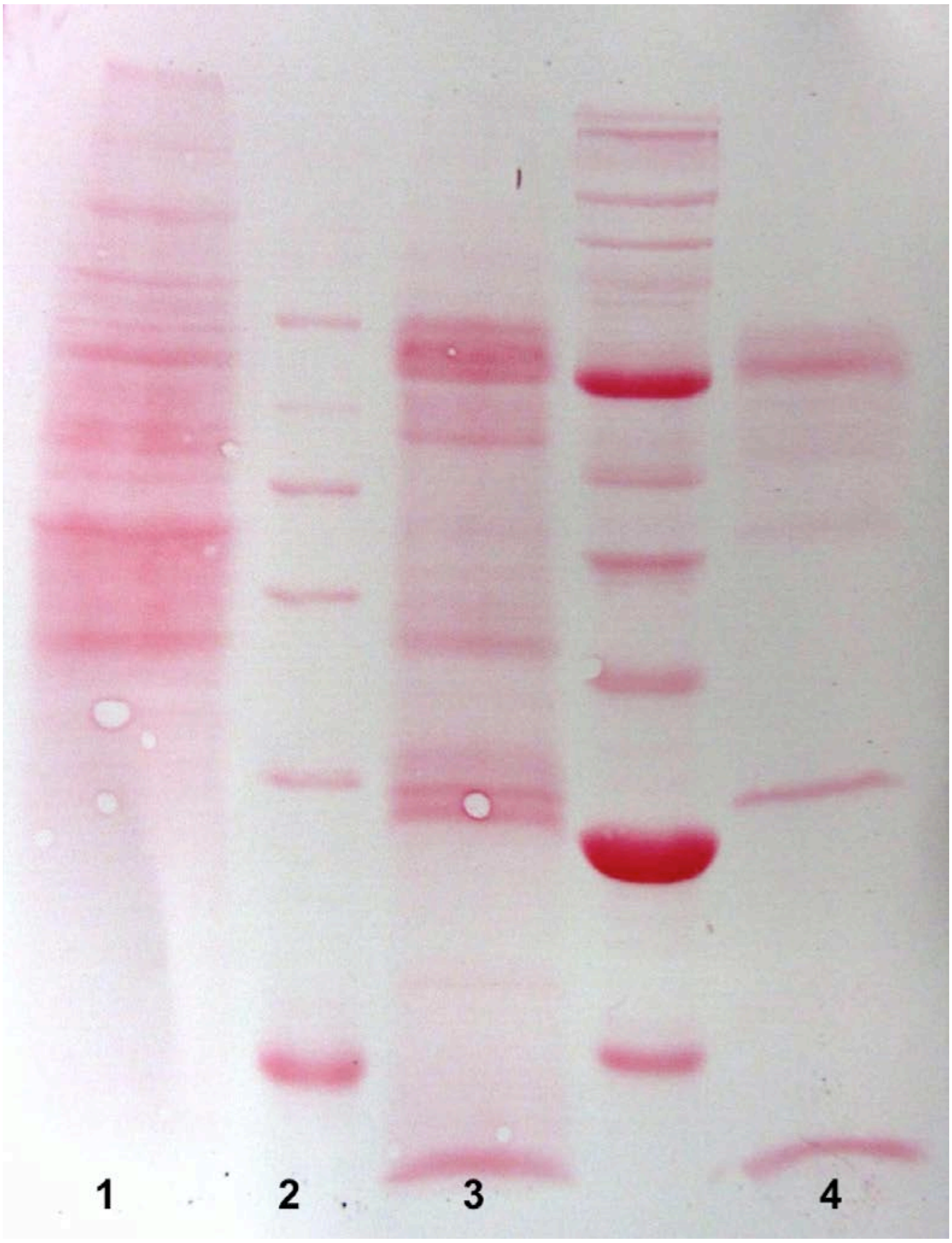

Original Ponceau S-stained PVFD-membrane shown in Figure 1. The lanes are numbered according to Figure 1 of the main manuscript. Lane 4 (plasmin control) has been replaced in the previously submitted version of Fig 1. This now corresponds exactly to the same PVDV-membrane as shown in lanes 1, 2 and 3.

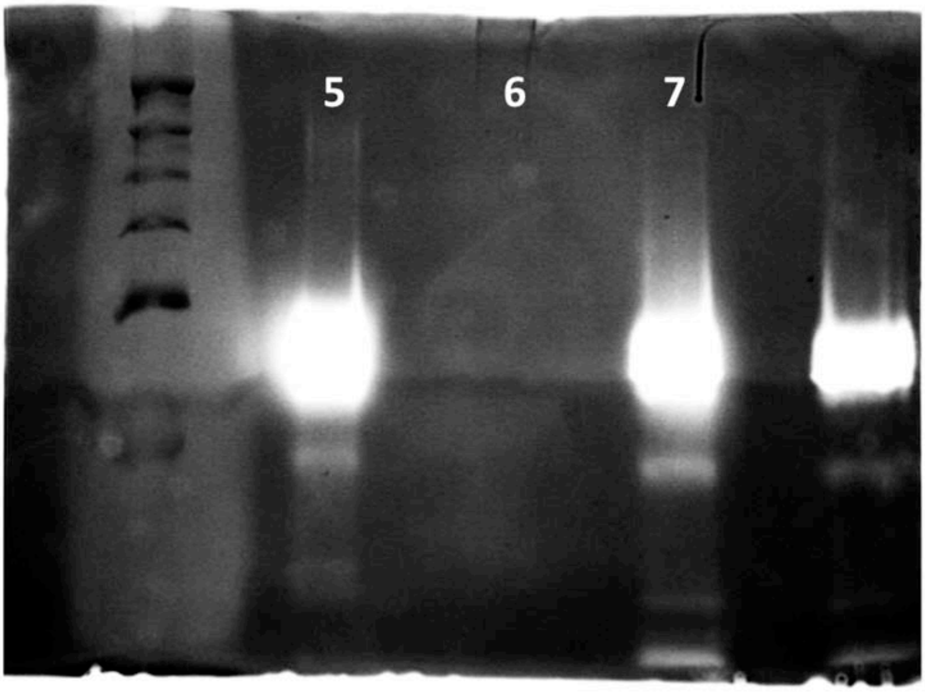

Original casein zymography gel shown in Figure 1 of the main manuscript. The zymography was difficult to photograph, due to the massive band produced by plasmin, which was used to activate pro-ovastacin. However, the band at the bottom of lane 7 of Fig. 1 is only observed in the presence of pro-ovastacin, not in the plasmin control (lane 5). Below are alternative image with different settings.

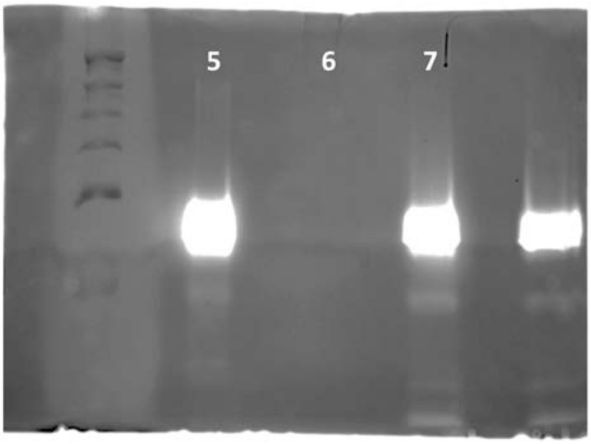

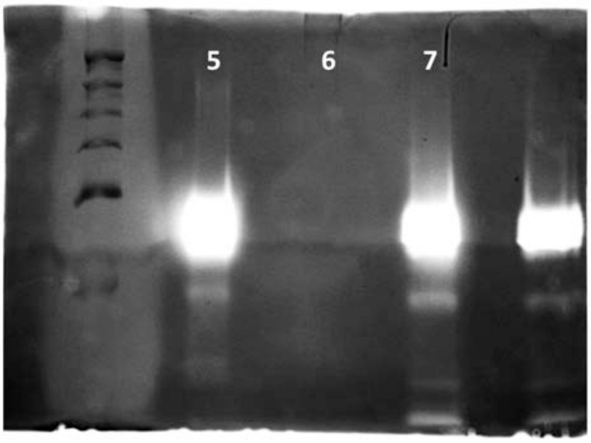

Supplement: Supplementary file 1 — Supplementary Figures and Information [file 41598_2018_37024_MOESM1_ESM.pdf]
